# Biocontrol of Bacterial Wilt Disease Through Complex Interaction Between Tomato Plant, Antagonists, the Indigenous Rhizosphere Microbiota, and *Ralstonia solanacearum*

**DOI:** 10.3389/fmicb.2019.02835

**Published:** 2020-01-10

**Authors:** Tarek R. Elsayed, Samuel Jacquiod, Eman H. Nour, Søren J. Sørensen, Kornelia Smalla

**Affiliations:** ^1^Institute for Epidemiology and Pathogen Diagnostics, Julius Kühn-Institut, Federal Research Centre for Cultivated Plants, Braunschweig, Germany; ^2^Department of Microbiology, Faculty of Agriculture, Cairo University, Giza, Egypt; ^3^Marine Microbiological Section, Department of Biology, Faculty of Natural and Life Sciences, University of Copenhagen, Copenhagen, Denmark; ^4^Agroécologie, AgroSup Dijon, INRAE, Université Bourgogne, Université Bourgogne Franche-Comté, Dijon, France

**Keywords:** *Ralstonia solanacearum*, biocontrol, latent infection, *fliC*, amplicon sequencing

## Abstract

*Ralstonia solanacearum* (biovar2, race3) is the causal agent of bacterial wilt and this quarantine phytopathogen is responsible for massive losses in several commercially important crops. Biological control of this pathogen might become a suitable plant protection measure in areas where *R. solanacearum* is endemic. Two bacterial strains, *Bacillus velezensis* (B63) and *Pseudomonas fluorescens* (P142) with *in vitro* antagonistic activity toward *R. solanacearum* (B3B) were tested for rhizosphere competence, efficient biological control of wilt symptoms on greenhouse-grown tomato, and effects on the indigenous rhizosphere prokaryotic communities. The population densities of B3B and the antagonists were estimated in rhizosphere community DNA by selective plating, real-time quantitative PCR, and *R. solanacearum*-specific *fliC* PCR-Southern blot hybridization. Moreover, we investigated how the pathogen and/or the antagonists altered the composition of the tomato rhizosphere prokaryotic community by 16S rRNA gene amplicon sequencing. *B. velezensis* (B63) and *P. fluorescens* (P142)-inoculated plants showed drastically reduced wilt disease symptoms, accompanied by significantly lower abundance of the B3B population compared to the non-inoculated pathogen control. Pronounced shifts in prokaryotic community compositions were observed in response to the inoculation of B63 or P142 in the presence or absence of the pathogen B3B and numerous dynamic taxa were identified. Confocal laser scanning microscopy (CLSM) visualization of the *gfp*-tagged antagonist P142 revealed heterogeneous colonization patterns and P142 was detected in lateral roots, root hairs, epidermal cells, and within xylem vessels. Although competitive niche exclusion cannot be excluded, it is more likely that the inoculation of P142 or B63 and the corresponding microbiome shifts primed the plant defense against the pathogen B3B. Both inoculants are promising biological agents for efficient control of *R. solanacearum* under field conditions.

## Introduction

The utilization of microbes to improve plant growth and health is gaining momentum. While significant knowledge on the links between plant traits and their microbiota was obtained from next generation sequencing technologies (Panke-Buisse et al., 2015), downstream applications of that knowledge are still difficult (Herrmann and Lesueur, 2013). Indeed, crop treatment with beneficial strains might be compromised by the poor survival rates of inoculants under field conditions (Dutta and Podile, 2010) and thus a better understanding of the ecology of inoculants is needed. Furthermore, deciphering the complex interaction of inoculants, pathogens, and the indigenous rhizosphere prokaryote community stand as one of the major challenges in understanding the ecology of plant–microbe interaction (Philippot et al., 2013; Shi et al., 2016; Berg et al., 2017).

*Ralstonia solanacearum* is a quarantine phytopathogen responsible for huge agricultural losses worldwide (Mansfield et al., 2012). *R. solanacearum* strains (Yabuuchi et al., 1995) form a species complex in the *Burkholderiaceae* family, divided into four phylotypes associated to geographic locations following human societies and agriculture expansion (I: Asia, II: America, III: Africa, IV: Pacific; Lowe-Power et al., 2018a, b). This soil-borne phytopathogen can infect more than 200 plant species, including crucial commercial crops. *R. solanacearum* survives for long periods in the environment (Graham et al., 1979; Grey and Steck, 2001) and when stressed (e.g., by cold temperatures; van Elsas et al., 2000, 2001; Kong et al., 2014), *R. solanacearum* initiates a resistance phase, the so-called “viable but non-culturable state” (VBNC), making it readily prone for dissemination *via* surface irrigation or infested soils. It may colonize rhizospheres of numerous non-host crops and weeds, or even hide under latent infection forms in endophytic compartments (Ciampi et al., 1980; van Overbeek et al., 2004). Although warm areas favor the development of the *R. solanacearum* wilting symptoms (Bocsanczy et al., 2014), cold-tolerant strains belonging to the brown rot phylotype IIB1 (Cellier and Prior, 2010) can infect host plants in temperate zones (Milling et al., 2009), making *R. solanacearum* a major threat to agriculture worldwide.

Infection is initiated through primary root tissue penetration *via* wounds or naturally occurring openings (e.g., secondary root emergence spots), followed by aggressive colonization of the host plant root system before becoming systemic, with appearance of typical shoot symptoms (Lowe-Power et al., 2018a, b). Several factors may influence *R. solanacearum* virulence, including anoxic condition. Indeed, while preferring oxygen, nitrate assimilation and respiration can enhance *R. solanacearum* attachment to the roots and promote its virulence (Dalsing and Allen, 2014; Dalsing et al., 2015). Furthermore, *R. solanacearum* persistence and success is ensured by efficient responses through up-regulation of genes involved in: (i) response to root exudates and low-oxygen conditions in rhizospheres (Colburn-Clifford and Allen, 2010), (ii) degradation pathways against plant defense compounds (e.g., hydroxycinnamic acid) (Lowe et al., 2015), or (iii) the adaptation to the nutrient-deprived xylem environment (Brown and Allen, 2004; Jacobs et al., 2012). For review on the topic, see Lowe-Power et al. (2018a, b).

Different strategies were developed to control *R. solanacearum*, such as agrochemicals, soil disinfection, antibiotics, antimicrobial plant extracts, resistant cultivars, genetic modification, crop rotations, organic amendments, lytic bacteriophages, and bacterial antagonists (reviewed by Yuliar et al., 2015). The use of environmentally-friendly biocontrol strategies relying on bacterial inoculant strains to enhance the soil wilt suppressiveness and plant priming capacity is a promising strategy, particularly in areas where the pathogen is endemic (Xue et al., 2013).

The major objective of this study was to assess the rhizosphere competence, the efficiency of reducing bacterial wilt symptoms on tomato, and the effects on the indigenous rhizosphere communities under greenhouse conditions for the two strains *Bacillus velezensis* (B63) and *Pseudomonas fluorescens* (P142). Seed inoculation and drenching was done, and tomato plants were grown in soil infested with *R. solanacearum* B3B or not. An integrative approach coupling several methods was employed to investigate pathogen abundance, rhizocompetence of the inoculant strains, root colonization patterns of the *gfp*-tagged P142, and the treatment effects on the rhizosphere prokaryotic communities. We hypothesized that priming of tomato plants against *R. solanacearum* is achieved through a complex interplay between plant, inoculants, rhizosphere microbiome shifts, and the pathogen.

## Materials and Methods

### Plant Materials and Bacterial Isolates

Tomato plant (*Lycopersicon esculentum* Mill. cv. Money maker) was selected as a host plant susceptible to *R. solanacearum* (strain B3B, race 3 biovar 2). The two antagonists, *B. velezensis* (B63) and *P. fluorescens* (P142), were selected after a pre-screening of *in vitro* antagonists on tomato plants for the greenhouse experiments reported here. Strains P142 and B63 were isolated from the tuber endosphere of potato plants grown in Germany or Egypt, respectively. The genomes of both strains were recently sequenced and the taxonomic assignment is based on multi-locus sequence analysis (Elsayed unpublished).

### Generation of Rifampicin Resistance Mutations and/or *gfp*-Tagged Antagonists

Rifampicin-resistant mutants (Rif^r^) were generated for both antagonists by inoculating 100 μl of 24-h bacterial culture of each antagonist onto R2A medium supplemented with rifampicin (50 μg/ml) and incubated (28°C). Rifampicin-resistant colonies were picked after 72 h and preserved at −80°C in LB broth medium supplemented with 20% glycerol. The Rif^r^ strain P142 was tagged with *gfp* gene encoding the green fluorescent protein (GFP) in a triparental mating (Haagensen et al., 2002). In brief, *Escherichia coli* CC118λpir was used as a donor for IncQ plasmid pSM1890 carrying the mini-Tn*5*-PA1/04/03-*gfp*mut3 cassette coding for the GFP as well as streptomycin (Sm^r^) and gentamicin (Gm^r^) resistance, *E. coli* CM544 carrying IncP-1β plasmid as a helper (Haagensen et al., 2002) and P142 as recipient. The presence of the *gfp* gene in P142 was tested by real-time PCR (Hajimorad et al., 2011) and the identity of the *gfp*-tagged antagonists was confirmed *via* comparing the BOX-fingerprints with the corresponding original isolate (Rademaker and De Bruijn, 1997). Antagonistic activity was re-tested for the *gfp*-tagged and/or Rif^r^ strain P142 according to Xue et al. (2013). Primers, PCR conditions, and probes used are compiled in Supplementary Table S1. The Rif^r^ B63 strain was not *gfp*-tagged as the IncQ plasmid pSM1890 could not stably replicate in B63.

### Rhizosphere Competence and Biocontrol Efficiency

**FIGURE 1:**
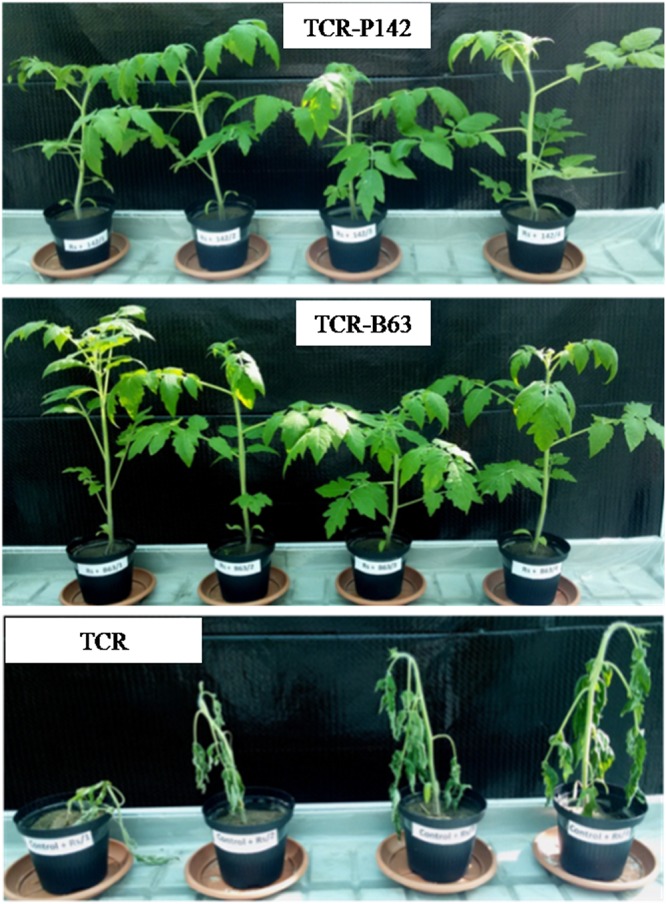
Wilting symptoms on tomato plants 14 days post transplantation inoculated with P142 or B63 compared to the pathogen control. TCR-P142, TCR-B63; TCR, control plants infected with *Ralstonia solanacearum* 1.78 10^6^ CFU/g soil.

The Rif^r^ antagonists P142 and B63 were grown in 50 ml LB-broth medium supplemented with corresponding antibiotics in an Erlenmeyer flask and incubated in a rotary shaker at 28°C. Bacterial cells were harvested by centrifugation (4500 × *g* for 10 min) after 24 h, pellets were washed three times (sterile NaCl 0.85%) and the density of the resuspended cells was adjusted to OD_600_ = 1.0 (about 10^8^ CFU/mL in NaCl 0.85%). Tomato seeds were soaked in the bacterial suspensions (20°C, 15 min) and air-dried (10 min). Inoculated and non-inoculated seeds were sown in diluvial sand soil (DS; information on the bacterial community composition and the physicochemical composition were reported by Schreiter et al., 2014) soil mixed with a standard potting soil (1:1 v/v) and kept in a greenhouse (2 weeks, 16 h light, 28°C). Uniformly developed seedlings were transferred to 15 cm pots filled with 300 g DS soil (four replicates per isolate, one plant per pot) under the same conditions. An additional drenching step was done one day prior to transplantation [14 days post sowing (dps)] with 4 ml bacterial culture suspension OD_600_ = 1.0 (about 10^8^ CFU/ml, Colony Forming Unit). Four plants treated with 4 ml saline solution served as control. Inoculated seedlings were transplanted to soil artificially infested by *R. solanacearum* B3B (TCR-B63; TCR-P142) or to control soil which was not infested (TC-B63; TC-P142). In addition, seedlings not inoculated with antagonists grown in non-infested soil served as control (TC), and as pathogen control (TCR) when grown in infested soil. Two different doses of *R. solanacearum* B3B were used, at a final population of 4.4 10^4^ (low dose) or 1.8 10^6^ CFU g^–1^ of soil (high dose). Only non-inoculated plants grown in soil infested with the high dose developed wilting symptoms (Figure 1). Symptoms were recorded daily for 2 weeks post transplanting. Hence, the analysis of rhizocompetence, biocontrol efficiency, and prokaryotic community analysis was done only for tomato plants grown in high dose B3B-infested soils. Fourteen days after transplanting, tomato plants were harvested and rhizosphere samples were obtained and analyzed as described below.

### Sampling and Sample Processing

Tomato plants were sampled 14 days after transplanting. Rhizosphere samples: the entire root system with the tightly adhering soil was transferred into a Stomacher bag, resuspended in 15 ml of 0.85% NaCl and treated with a Stomacher 400 Circulator (Seward Ltd., Worthing, United Kingdom) at middle speed. The supernatant was collected and the Stomacher treatment was repeated twice. A total of 45 ml of supernatant was collected in 50 ml Falcon tubes and used for plating and harvesting the rhizosphere cell pellet after centrifugation. To sample the endorhiza communities, the same root used for the rhizosphere analysis was surface-sterilized by dipping the root in sodium hypochlorite (5% active chlorine) for 3 min, followed by 3 min in hydrogen peroxide 3% according to Sturz et al. (1999), then three washing steps for 10 min each using sterilized saline. The root sterility was checked by pressing the roots on R2A medium. Surface-sterilized roots were blended using sterilized mortar and pestle. Serial dilutions were prepared from the rhizosphere and endorhiza bacterial suspension and plated onto King’s B Agar medium (King et al., 1954), supplemented with Rif^50^, Sm^50^, Gm^10^, ampicillin^100^, chloramphenicol^30^, and cycloheximide^100^ (Cyc) for P142, and PCA medium supplemented with Rif and Cyc for B63. CFU counts were enumerated after 48 h of incubation at 28°C and related to gram root fresh weight (rfw). The CFU counts of B3B were determined using semi-selective medium from South Africa (SMSA) supplemented with suitable antibiotics as described by Engelbrecht (1994). CFU counts were recorded after 48 h incubation at 28°C. Significant differences of the CFU counts were analyzed by Tukey’s LSD test at (*p* ≤ 0.05) using SAS software.

Total community DNA was extracted from the rhizosphere pellets (500 g) with the FastDNA spin kit for soil (MP Biomedicals, Heidelberg, Germany). The GENECLEAN SPIN Kit (MP Biomedicals, Heidelberg, Germany) was applied to purify the extracted DNA according to the manufacturer’s instructions. DNA samples were diluted 1:10 by 10 mM Tris HCl pH 8.0 and stored at −20°C for further analysis.

### Confirmation of the *in planta* Biological Control of *Ralstonia solanacearum* and Latent Infection

The two antagonists P142 and B63 were tested in a second greenhouse experiment with more plants to confirm the results of the previous greenhouse experiment and to test for latent infection. Tomato seeds were treated with each antagonistic isolate culture suspension (OD_600_ = 1.0), respectively. Seeds were germinated and grown in potting soil for 1 month; before transplanting, a drenching with 4 ml of each antagonist (OD_600_ = 1.0) was applied 28 dps, control plants were treated with the same volume of NaCl 0.85%. Seedlings were transferred to pots filled with 300 g B3B-infested DS soil (32 replicates each) or non-infested soil. Untreated plants served as control. The soil was artificially infested with 4 ml B3B per pot (OD_600_ = 1.0) to a final density of 1.3 10^6^ CFU g^–1^ of soil. The development of wilting symptoms was daily observed for one month. After 14 days, four tomato plants were harvested, rhizosphere samples were processed, and CFU counts of B3B, P142, and B63 were determined as described above. In addition, surface-sterilized tomato shoots (in sodium hypochlorite 5% for 3 min, followed by 3% H_2_O_2_ for additional 3 min, and finally three washing steps in sterile water) were immediately frozen in liquid nitrogen and ground in sterilized mortar and pestle, then ground plant materials were kept at −80°C for total community DNA extraction.

### Real-Time PCR-Based Quantification of Target Genes From the Rhizosphere Total Community DNA

Bacterial 16S rRNA gene copies (*rrn*) were estimated in rhizosphere community DNA according to Suzuki et al. (2000). The copy numbers of *gfp* gene were determined in rhizosphere total community DNA of TCR-P142 and TC-P142-treated plants and related to 16S rRNA gene copies (Yankson and Steck, 2009). Primers targeting the UDP-3-O-acyl-GlcNAc deacetylase, proposed by Chen et al. (2010), were modified in order to improve the specificity for B3B (Supplementary Figure S1). The copy numbers of *R. solanacearum* B3B were quantified in total community DNA using the modified primers under the following conditions: 95°C for 2 min followed by 40 cycles at 95°C for 20 s, 62°C for 25 s, 72°C for 35 s, and finally 80°C for 3 s before plate read, a melt curve step was included to verify the primer specificity. The primer pairs (B3B-RSF and B3B-RSR) were used under PCR conditions of 94°C for 5 min, and 30 cycles of 94°C for 1 min, 54°C for 1 min and 72°C for 1 min, and then 10 min at 72°C were applied before cooling down to 4°C, to amplify a fragment of 441 bp from B3B which was subsequently cloned in *E. coli* using the pGEM-T Easy Vector system I (Promega Corporation, Madison, WI, United States) according to the manufacturer’s protocol. The pGEM-T Vector was re-extracted using the GeneJET Plasmid Miniprep kit (Thermo Fisher Scientific, Vilnius, Lithuania) and used for serial dilutions to establish the standard calibration for the real-time PCR. All primers are listed in Supplementary Table S1.

### Illumina Sequencing and Analysis of 16S rRNA Gene Amplicons From Total Community DNA

Amplicon sequencing was performed according to defined and acknowledged best practices as previously described (Schöler et al., 2017). Prior to tag-encoded 16S rRNA gene sequencing, the 24 samples of extracted DNA were subjected to an initial PCR amplification step using a set of primers, 341F and 806R (Supplementary Table S1), which flank the approximately 460 bp variable V3–V4 region of the 16S rRNA gene of the target group Prokaryotes including domains of Bacteria and some Archaea. A second amplification step of the corresponding 16S rRNA gene region using the same primers with attachment of adaptors and barcode tags was done as previously described (Jacquiod et al., 2017). Purification and size selection (removal of products of less than 100 bp) of the approximately 620 bp PCR amplicon products was performed using Agencourt AMPure XP beads (Beckman Coulter, Brea, CA, United States) according to the manufacturer’s instructions. The concentration of purified amplicon samples was subsequently measured using a Qubit Fluorometer (Life Technologies, Carlsbad, CA, United States), the samples were pooled and adjusted to equimolar concentrations, concentrated using the DNA Clean and Concentrator^TM^-5 kit (Zymo Research, Irvine, CA, United States), and finally subjected to 2 × 250 bp paired-end high-throughput sequencing on an Illumina® platform (Illumina, San Diego, CA, United States).

Amplicon sequences were analyzed using qiime_pipe^1^ with default settings, which performs sample demultiplexing, quality-based sequence trimming, primer removal, and paired-end reads assembly prior to annotation workflow (Caporaso et al., 2010). Paired-end mating was applied with a minimum overlap length of 50 bp, maximum mismatches of 15, and a minimum quality of 30. Criteria for sequence trimming were based on: (1) reads shorter than 200 bp, (2) average quality scores lower than 25, (3) maximum number of ambiguous bases, and (4) six as maximum lengths of homopolymers. Chimera check was done with UCHIME (Edgar et al., 2011) and operational taxonomic units (OTUs) were picked at 97% sequence identity level. OTU representative sequences were selected by the highest abundance within the cluster and assigned to taxonomy using the RDP classifier (Cole et al., 2003), with a confidence threshold of 80%. Information regarding the sequence counts for each sample is provided in the Supplementary Table S2, and rarefaction curves are presented in Supplementary Figure S2. Community-level analysis was performed with a cluster dendrogram using the unweighted pair group method with arithmetic mean (UPGMA, Euclidean distance). Significant changes in the relative abundance of dominant taxa were identified with an ANOVA under a generalized linear model, followed by Tukey’s honest significance detection test (*p* < 0.05). Sequences were submitted for deposition at the public repository Sequence Read Archive (SRA^2^) with the accession number PRJNA574588^3^.

### PCR-Southern Blot Hybridization-Based Detection of *R. solanacearum* Specific *fliC* Gene

PCR amplification with primers targeting *Rs*-*fliC* gene was performed according to Schönfeld et al. (2003) from total community DNA from rhizosphere and shoots of tomato plants grown in B3B-infested soils. PCR products were analyzed by 1% agarose gel electrophoresis for 1 h (50 V), gels were checked by UV light after staining with ethidium bromide and Southern-blotted as described by Binh et al. (2008). Hybridization was performed with Digoxygenin-labeled *fliC* probe generated from purified PCR products obtained with B3B by means of the DIG DNA labeling kit (Roche Applied Science, Mannheim, Germany).

### Confocal Laser Scanning Microscopy (CLSM) Analysis

Tomato roots were analyzed by confocal laser scanning microscopy (CLSM) 5 days after drenching with *gfp*-tagged isolate P142 to detect and localize its colonization of tomato ecto- and endophytic root compartments. The tightly attached soil particles were removed by shaking the root vigorously, then cut into small pieces of ca. 2 cm, and mounted with a few drops of 0.85% NaCl. Root pieces were analyzed using Leica TCS SP2 CLSM. Argon/Krypton laser (excitation at 488 nm) was used to detect the excitation of the GFP combined with the transmitted light pictures. Detected GFP signals were confirmed by applying lambda scan.

## Results

### Rhizocompetence of the Inoculant Strains

**FIGURE 2:**
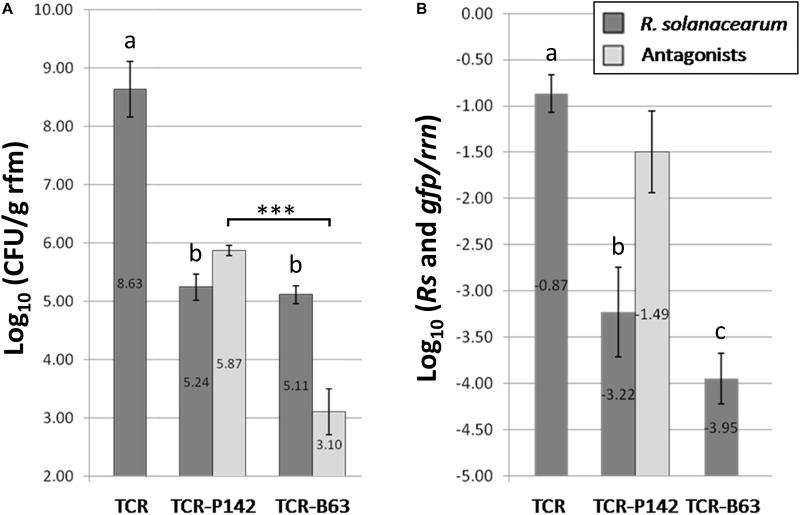
**(A)** Significant reduction of Log_10_ (colony forming units/g root fresh mass) for *R. solanacearum* B3B (dark gray) for inoculated treatments with the antagonists TCR-P142 and TCR-B63 14 days post transplantation compared to the pathogen control TCR as indicated by lower case letters (Tukey’s HSD, *p* < 0.05). In light gray bars the Log_10_ (colony forming units/g root fresh mass) of P-142 and B63 are given. The significant differences in the CFU counts of both antagonists are indicated by “***” (Tukey’s HSD, *p* < 0.05). **(B)** A significant reduction of Log_10_ (target gene/rrn) for *R. solanacearum* B3B (dark gray) was also shown by real time quantitative PCR for *R. solanacearum* (dark gray) related to 16S rRNA gene (*rrn*) copy numbers 14 days post transplanting (Tukey’s HSD, *p* < 0.05). The light gray bar represents the Log_10_ (*gfp*/rrn) for P142. No quantitative PCR data four B63 as *gfp* labeling was not successful for B63.

The potential of the two antagonists to colonize the rhizosphere of tomato root, expressed by means of CFU counts, was determined 14 days after transplanting. The CFU counts of P142 indicated efficient rhizosphere colonization with 5.9 Log_10_ CFU g^–1^ rfm, while rather low CFU counts were detected for B63 with 3.1 Log_10_ CFU g^–1^ rfm (see Figure 2A).

The high relative abundance of the *gfp*-copy number (−1.49) determined by qPCR in total community DNA confirmed the high rhizosphere competence of P142 (Figure 2B). Noteworthy, the relative abundance of P142 in the rhizosphere was significantly higher for P142-treated plants grown in B3B-infested soils (−1.49 Log_10_ *gfp* copy number/16S rRNA gene g^–1^ g of rfm) compared to non-infested soils (−2.95 Log_10_ *gfp* copy number/16S rRNA gene g^–1^ of rfm) (Figure 2B).

### Biological Control of *Ralstonia solanacearum in planta*

For assessing the efficiency of P142 and B63 to reduce tomato wilt symptoms, plants transplanted into soil infested with the high dose of B3B were assessed daily for the appearance of wilting symptoms. All tomato control plants grown in soil infested with high B3B dose (TCR; 1.8 10^6^ B3B CFU g^–1^ soil) had collapsed 14 days post infection (dpi), while no uniform wilting symptoms were observed when the soil was infested with low B3B population density (4.4 10^4^ CFU g^–1^ soil). Thus, effects of P142 and B63 on the indigenous prokaryotic communities of the tomato rhizosphere were only assessed for the plants grown in soil with the high B3B density. Plants inoculated with P142 or B63 showed no wilting symptoms 14 dpi (Figure 1). The tomato plants treated with antagonists showed significantly lower B3B CFU counts compared to the TCR (8.6 Log_10_ CFU g^–1^ root). Approximately three orders of magnitude lower B3B CFU counts were recorded for both TCR-P142 and TCR-B63 (5.2 and 5.1 Log_10_ CFU g^–1^ rfm, respectively; Figure 2A). The relative abundance of B3B was very high in the pathogen controls (TCR: -0.85 Log_10_ copy number/16S rRNA gene g^–1^ of rfm; Figure 2B) and significantly lower in DNA from the rhizosphere of inoculated tomato plants with relative abundance of B3B being reduced about two to three orders of magnitude in the treatments with P142 (−3.22 Log_10_ copies/rrn) and B63 (−3.95 Log_10_ copies/rrn) compared to TCR (Figure 2B).

For further confirmation, the *R. solanacearum-*specific *fliC* gene was detected by PCR with subsequent Southern blot hybridization. Very strong hybridization signals were obtained for TCR rhizosphere samples of tomato plants grown in soil infested with the high *R. solanacearum* population compared to the rhizosphere samples grown in soil infested with low densities (positive signals for three replicates out of four) (Supplementary Figure S3), while no hybridization signals were detected in the uninfected control samples (TC). The *fliC* hybridization patterns obtained from the rhizosphere samples concurred with results of *R. solanacearum* real-time qPCR data, as stronger hybridization signals corresponded to high relative abundance of *R. solanacearum* detected *via* qPCR. Weak or no hybridization signals were detected for TCR-B63 followed by TCR-P142.

### Biological Control of *R. solanacearum*: Checking for Latent Infections

**FIGURE 3:**
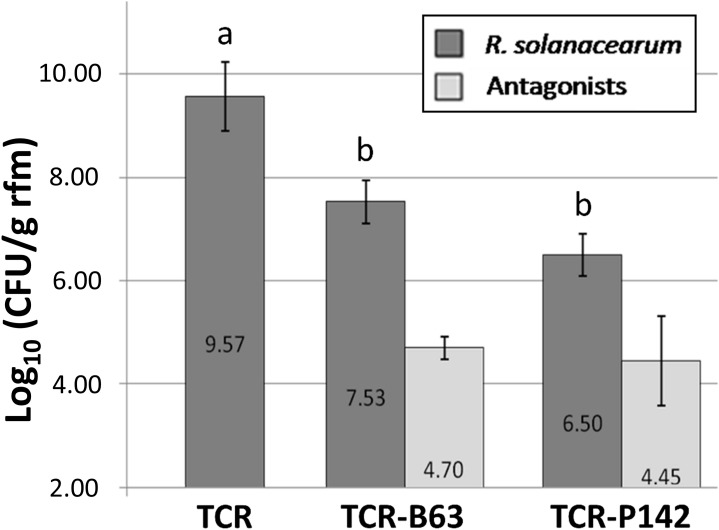
Significant reduction of the Log_10_ (colony forming units/g root fresh mass) for *R. solanacearum* B3B (dark gray) for inoculated treatments with the antagonists TCR-P142 and TCR-B63 of the second greenhouse experiment 14 days post transplantation compared to the pathogen control TCR as indicated by lower case letters (Tukey’s HSD, *p* < 0.05). In light gray bars the Log_10_ (colony forming units/g root fresh mass) of P-142 and B63 are given.

An additional greenhouse experiment with a larger number of plants was conducted in order to confirm the efficiency of both B63 and P142 antagonists against *R. solanacearum* and to check for latent infections. Wilting symptoms of inoculated and non-inoculated plants grown in soil infested with high *R. solanacearum* densities (3.9 10^6^ CFU g^–1^ of soil) was recorded 14 days post transplanting. Out of the total 32 replicates, 19 TCR plants (59%) had collapsed. The number of collapsed plants was similar in both TCR-P142 and TCR-B63, only six plants representing 18.8%. The CFU counts of the antagonists in the rhizosphere of symptomless plants were 4.7 and 4.5 Log_10_ (CFU/g rfm) for TCR-B63 and TCR-P142, respectively (Figure 3). While *R. solanacearum* populations reached 9.57 Log_10_ (CFU/g rfm) in TCR samples, significantly lower pathogen levels were recorded for P142 and B63-inoculated plants (Log_10_ (CFU/g rfm) root: 6.5 and 7.5, respectively).

**FIGURE 4:**
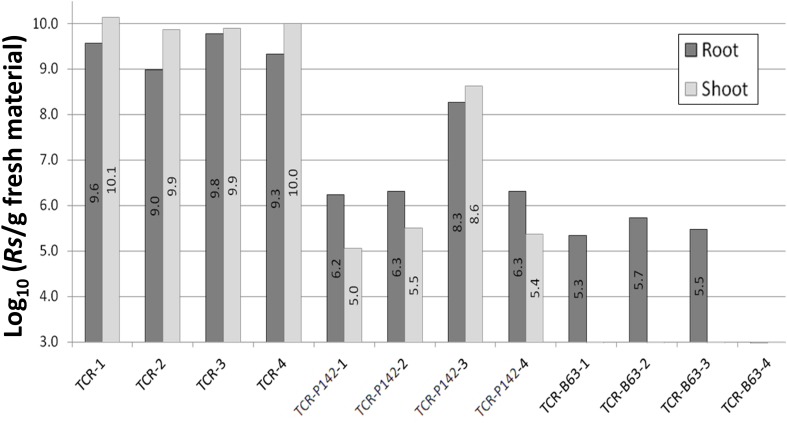
*Ralstonia solanacearum* gene copy numbers estimated in total community DNA from surface-sterilized tomato root and shoot samples by qPCR. Gene copy numbers are displayed as individual sample values for all replicates.

Quantification of the *R. solanacearum*-specific gene confirmed significantly lower B3B copy numbers in total community DNA of TCR-P142 and TCR-B63 (6.8 and 4.0 Log_10_ copies per g^–1^ of rfm, respectively) compared to TCR samples (Average number of TCR samples = 9.4 Log_10_ copies/g rfm) (Figure 4). Similarly, in tomato shoot samples, significantly lower B3B CFU counts were recorded in TCR-P142 antagonists (ranging from 5.0 to 8.6 copies per g^–1^ of sfm) compared to TCR samples (9.9–10.1 copies per g^–1^ of sfm) while it was below detection limit in shoot of tomato plants inoculated with B63. While the Log_10_ (gfp/rfm) gene copy number of *gfp g*^–1^ *rfw* was 7.9 ± 0.38 in the rhizosphere of P142-inoculated tomato plants (Figure 4), two out of four replicates showed, additionally, colonization of tomato shoot endophytic compartments [5.6 ± 0.7 Log_10_ (*gfp* copies *g*^–1^ sfm)].

**FIGURE 5:**
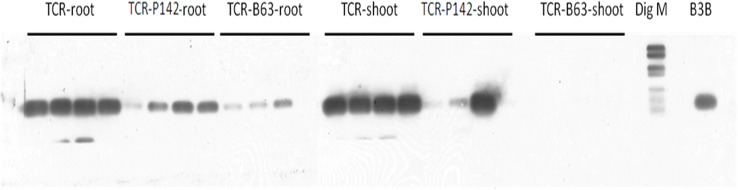
PCR-Southern blot hybridization of *fliC* gene specific for *R. solanacearum* in tomato rhizosphere and shoot total community DNA samples.

Southern blot hybridization targeting *fliC* gene in the total community DNA of both rhizosphere and shoot samples showed patterns concurred with the *R. solanacearum* real-time PCR results (Figure 5). Stronger hybridization signals were obtained for TCR rhizosphere samples compared to TCR-P142 and TCR-B63. Regarding the shoot samples, compared to the strong signals obtained for the TCR, only one strong and two very weak signals were detected in TCR-P142, while no hybridization signals were detected in the shoots of TCR-B63 plants.

### 16S rRNA Gene Amplicon Illumina Sequencing

Illumina amplicon sequencing of V3–V4 regions from the 16S rRNA gene was obtained from the rhizosphere of inoculated or non-inoculated tomato plants grown in B3B-infested or non-infested soil using 2 × 250 bp paired-end Illumina MiSeq. A total of 630,016 bacterial sequences were generated from six treatments (four replicates per each treatment; Supplementary Table S2 and Supplementary Figure S2). The highest number of bacterial sequences was detected for TCR-B63 and TC-B63 (27,865 and 32,112 sequences, respectively), while the lowest number of bacterial sequences was detected for TCR-P142 and TC-P142 (21,171 and 21,325 sequences, respectively). The TC and TCR samples had 32,988 and 31,044 sequences, respectively. The bacterial sequences were affiliated with 10 phyla, 29 classes, 56 orders, 135 families, and 263 genera.

### Tomato Rhizosphere Bacterial Community Composition

In the rhizosphere of healthy tomato plants (TC), Proteobacteria were the most dominant phylum with relative abundance of 62.3%, followed by Actinobacteria, Bacteroidetes, and Firmicutes (14.6, 12.7, and 8.4%, respectively). Other phyla were detected in the rhizosphere with relative abundance of less than 1% such as Gemmatimonadetes, Planctomycetes, Nitrospirae, Verrucomicrobia, and Chloroflexi. Among the Proteobacteria, most OTUs were affiliated to Alphaproteobacteria (relative abundance of 31.2%), followed by Gammaproteobacteria and Betaproteobacteria (17.6 and 13.1%, respectively), while the relative abundance of Deltaproteobacteria was low (0.4%) (Table 1). At the genus level, *Rhodanobacter* was the most dominant genus with relative abundance of 9% followed by *Shinella*, *Rhizobium*, *Arthrobacter*, *Massilia*, *Sphingobium*, *Sphingomonas*, and *Devosia* (Table 2).

**TABLE 1:** Relative abundance of dominant phyla and classes in the rhizosphere of tomato affected by the pathogen *R. solanacearum* and/or inoculation (average ± standard error of the mean, *n* = 4 per treatment).

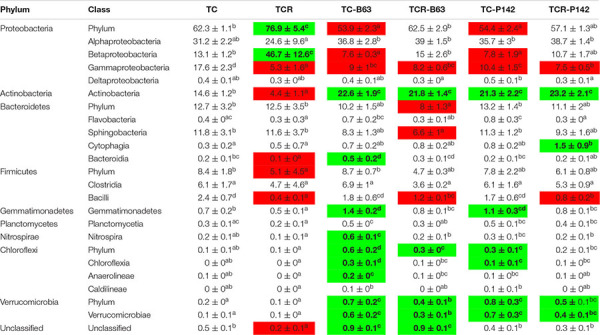

*Number shows the average percentage followed by ± standard deviation (*n* = 4). Treatments sharing the same letters are non-significantly different (*p* < 0.05, ANOVA under generalized linear model followed by Tukey’s Honest Significant Detection test). Significant increases in abundance compared to the TC are highlighted in green, while significant decreases are highlighted in red.*

**TABLE 2:** Relative abundance of dominant responding OTUs (relative abundance ≥ 0.5%) detected two weeks after inoculation and/or infection in the rhizosphere of tomato (average ± standard error of the mean, *n* = 4 per treatment).

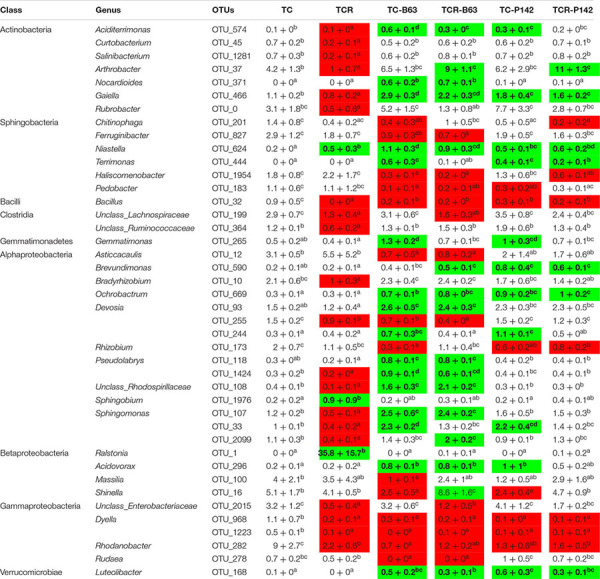

*Number shows the average percentage followed by ± standard deviation (*n* = 4). Treatments sharing the same letters are non-significantly different (*p* < 0.05, ANOVA under generalized linear model followed by Tukey’s Honest Significant Detection test). Significant increases in abundance compared to the TC are highlighted in green, while significant decreases are highlighted in red.*

### Inoculation and Infection-Dependent Prokaryotic Community Structure in Tomato Rhizosphere

**FIGURE 6:**
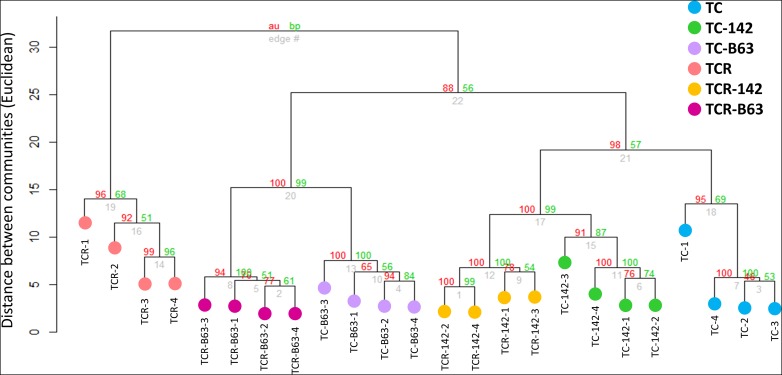
Cluster analysis of tomato rhizosphere bacterial communities responding to inoculation by antagonists and/or infection with *R. solanacearum* (clustering method = UPGM, distance = Euclidean, bootstraps = 1000).

Cluster dendrogram analysis (UPGMA) based on the relative abundance of all bacterial OTUs obtained from tomato rhizospheres revealed two major distinct clusters. The first included only the TCR samples, while the second combined the samples of TCR-B63, TCR-P142, TC-B63, and TC-P142 in addition to TC samples (Figure 6). Notably, the clustering of the four TCR replicates was correlated with the development of wilting symptoms, as TCR1 was the first plant showing wilting symptoms (4 days before harvest) followed by TCR2, while both TCR3 and TCR4 showed symptoms only 1 day before harvest. However, the second cluster was divided into two sub-groups based on inoculation, then each was further divided, attributed to the presence of B3B, forming a total of four separate clusters (TC-B63 and TCR-B63; TC-P142, TCR-P142, and TC).

### Influence of *R. solanacearum* on the Prokaryotic Community Composition of Tomato Rhizosphere

*Ralstonia solanacearum* B3B strongly shaped the bacterial community composition in the rhizosphere of tomato plants (TCR). Significant changes in the relative abundance of dominant taxa were identified by Tukey’s honest significance test under a generalized linear model. The analysis of rhizosphere microbiota of tomato plants grown in B3B-infested soil (TCR) was compared with the non-infected control plants (TC). The results showed that the pathogen massively dominated the rhizosphere microbiota. Thus, the relative abundance of Betaproteobacteria increased (46.7 ± 12.6%, mostly *Ralstonia*) compared to healthy non-infected tomato plants (13.1 ± 1.2%). In contrast, Gammaproteobacteria, Actinobacteria, Bacteroidetes, and Firmicutes (particularly *Bacilli*) decreased in DNA from TCR (Table 1). At OTU level, a decreased relative abundance was detected for 53 taxa, affiliated with 39 different genera. *Rhodanobacter*, *Dyella*, *Arthrobacter*, *Rubrobacter*, *Sphingomonas*, *Bradyrhizobium*, *Curtobacterium*, *Salinibacterium*, and *Bacillus* showed the highest decrease. Besides *Ralstonia*, OTUs affiliated with *Sphingobium* and *Niastella* increased in TCR compared to TC (Table 2 and Supplementary Tables S2, S4).

### Inoculation of *Bacillus velezensis* B63 or *Pseudomonas fluorescens* P142 Antagonists Changed the Tomato Rhizosphere Bacterial Community Composition

In the rhizosphere of tomato plants grown in non-infested soil and inoculated with either B63 or P142 (TC-B63; TC-P142), a decrease in Proteobacteria, especially Betaproteobacteria and Gammaproteobacteria classes, was observed (Table 1). The phyla Actinobacteria, Gemmatimonadetes, Chloroflexi, and Verrucomicrobia increased in TC-B63 and TC-P142 samples. At OTU level, the highest number of responders was detected in TC-B63 followed by TC-P142. In TC-B63 rhizosphere DNA, a total of 116 OTUs changed, 85 OTUs increased while 31 OTUs decreased. For TC-P142, a total of 68 OTUs changed, 52 OTUs increased while 16 OTUs decreased (Table 2 and Supplementary Table S4). A total of 56 OTUs commonly responded with similar patterns with either TC-B63 or TC-P142 (44 increased and 12 decreased responders). Regarding the strong responders (OTUs that increased or decreased more than two folds compared to TC), members from Alphaproteobacteria substantially increased (*Ochrobactrum*, *Devosia* OTU-93 and OTU-244, *Pseudolabrys*, *Rhodospirillaceae*, and *Sphingomonas*), except in the genus *Rhizobium* (for both antagonists), as well as *Asticcacaulis* and *Devosia* in TC-B63 which all decreased. OTUs affiliated to Gammaproteobacteria (*Rhodanobacter* and *Dyella*) decreased in both TC-B63 and TC-P142, while *Rudaea* decreased only in TC-B63. However, within the same class and/or genus, different OTUs showed variable responses to the inoculation with antagonists, as abundances of some OTUs increased while others decreased, compared to the TC samples (Table 2).

### The Complex Interaction Between *R. solanacearum*, Antagonists, and Indigenous Rhizosphere Microbiota

Sequences affiliated to *Ralstonia* were about three orders of magnitude lower in TCR-P142 as well as in TCR-B63 compared to TCR. Thus, the relative abundance of B3B was only 0.1% in both TCR-B63 and TCR-P142 while it reached 35.8% in the TCR samples. Alpha-diversity analysis on the rhizosphere microbiota revealed that all tested indices were decreased when tomato plants grew in B3B-infested soil (TCR) due to the dominance of *Ralstonia* (Supplementary Table S3). Richness and evenness were higher when tomato plants grown in non-infested soil were inoculated with B63 (TC-B63; Supplementary Table S3).

Gammaproteobacteria and *Bacilli* were lower in both TCR-P142 and TCR-B63 compared to TC-P142 and TC-B63, respectively. Bacteroidetes, particularly Sphingobacteria, were lower in TCR-B63 compared to TC-B63 samples. The phyla Actinobacteria and Verrucomicrobia showed a higher relative abundance in both TCR-B63 and TCR-P142 samples compared to TC-B63 and TC-P142 (Table 1). At OTU level, the abundance of 90 responders changed in TCR-B63 samples (57 increased and 43 decreased), while 54 OTUs changed in TCR-P142 (35 increased and 19 decreased) compared to TC-B63 and TC-P142. A total of 35 OTU responders were shared between both TCR-B63 and TCR-P142 (21 increased and 14 decreased) (Table 2). OTUs affiliated to *Arthrobacter*, *Gaiella*, *Niastella*, and *Ochrobactrum* had a higher relative abundance in both TCR-B63 and TCR-P142, while *Devosia*, *Shinella*, *Sphingomonas*, *Acidovorax*, and OTUs affiliated with unclassified *Rhodospirillaceae* were higher only in TCR-B63. *Bacillus*, *Dyella*, and *Rhodanobacter* were lower in both TCR-B63 and TCR-P142 while those of *Rhizobium* and *Chitinophaga* decreased only in TCR-P142 (Table 2 and Supplementary Table S4).

### Localization of *gfp*-Tagged P142 in Rhizosphere and Root Endophytic Compartments

**FIGURE 7:**
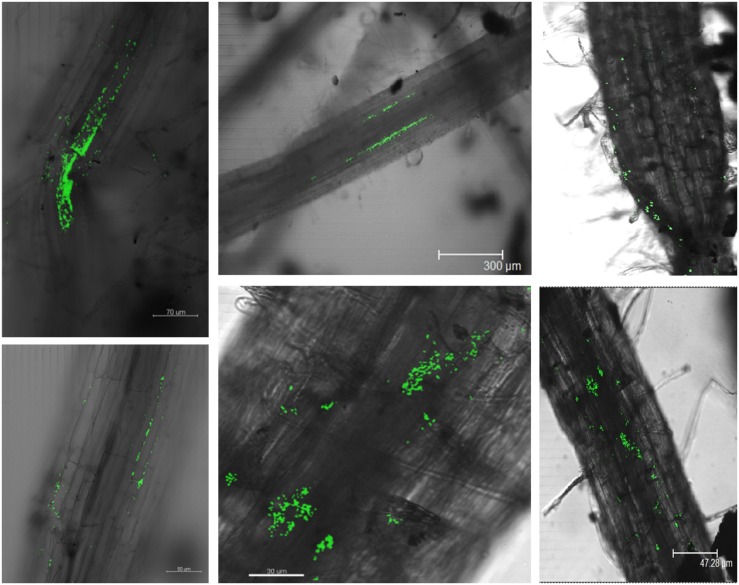
CLSM confirms the colonization of tomato roots and root endophytic compartments by the *gfp*-tagged P142 (5 days post drenching). Root pieces were analyzed using Leica TCS SP2 CLSM. Argon/Krypton laser (excitation at 488 nm) was used to detect the excitation of the GFP combined with the transmitted light pictures.

Confocal laser scanning microscopy was used to obtain insights into the P142 root colonization patterns. Tomato root surfaces were efficiently colonized by *gfp*-tagged biocontrol bacteria. Strong signals were detected five days after drenching in inoculated plants while no signals were detected in control plants (besides auto-fluorescence that could be removed by narrowing the detection wavelength based on lambda scan). The *gfp*-tagged strain P142 was detected in lateral roots as well as in root hairs. Micro-colonies were observed along the root surface while the endophytic life style of P142 was confirmed by the colonization and invasion of epiphytic root cells as well as xylem vessels (Figure 7).

## Discussion

Here we investigated the efficacy of two *in vitro* antagonists of *R. solanacearum* to reduce wilting symptoms in tomato plants under greenhouse conditions and followed the abundance of the inoculant strains and the pathogen using cultivation-dependent and independent methods. Field testing was no option as *R. solanacearum* is a quarantine organism. Strain P142 showed good survival in the rhizosphere of tomato plants. By means of CLSM, we could show that P142 was able to colonize tomato roots internally without adverse symptoms, indicating good potential for being true endophytes. Surprisingly, although strain B63 seems to have a rather weak rhizosphere competence its efficiency to reduce wilting symptoms and the abundance of B3B in the rhizosphere and in the tomato shoots was remarkable. Also the numbers of taxa with increased or decreased relative abundance in response to the inoculation of B63 was higher compared to P142. Inoculation of B63 strongly shaped the rhizosphere community in TC-B63 and TCR-B63. One possible explanation is that B63 colonized the soil fraction not that close to the root and thus was missed with the rhizosphere sampling protocol used. In addition, in the present study, vegetative B63 cells were inoculated and not spores, as typically done for *Bacillus* inoculants. Based on pre-experiments, we have used seed inoculation and drenching before transplanting, as recommended also by Götz et al. (2006).

A high B3B population density (1.78 10^6^ g^–1^ soil) was required to observe uniform infection, and maximum symptom severity was recorded when B3B populations reached 8.44 Log_10_ (CFU). The CFU counts of B3B in the rhizosphere were higher in the second greenhouse experiment, and this might explain the appearance of wilting symptoms despite a two to three orders of magnitude reduction of the B3B CFU counts in the plants treated with B63 or P142. Results confirmed that pathogenicity regulation is density-dependent (Schell, 2000), requiring high cell concentration for triggering virulence gene expression and accumulation of deleterious metabolites to cause acute infection (e.g., putrescine; Lowe-Power et al., 2018a). Meanwhile, although a significant decrease in wilted plants (18.8 *vs* 59%) and lower *R. solanacearum* densities were observed with both inoculants, only shoot endophytic compartments of TCR-B63 exhibited no latent infection. Therefore, B3B detection in asymptomatic plants showed that absence of visible symptoms of the disease is not a reliable proxy for pathogen eradication. This has important implications for trade with countries where *R. solanacearum* is endemic. However, it remains unclear at which density level expression of pathogenicity determinants and the subsequent development of wilting symptoms occur, as published studies are often done under control conditions where *R. solanacearum* densities are high (∼10^9–10^; Lowe-Power et al., 2018a). In the second greenhouse experiment, higher CFU counts for B3B (9.57 Log_10_ (CFU/g rfm); Figure 3) were detected in the rhizosphere compared to the first greenhouse experiment (8.63 Log_10_ (CFU/g rfm); Figure 2A), and thus the detection of some plants with wilting symptoms for TCR-P142 and TCR-B63 compared to no wilting in the first greenhouse experiment was not too surprising. Several mechanisms are likely at play to explain the drastic reduction of B3B abundance and the absence of wilting symptoms in the first greenhouse experiment. Plant systemic resistance induction was investigated by Park et al. (2007) for *R. solanacearum* biological control *via Bacillus vallismortis* strain Iq EXTN-1, but this aspect is going beyond the scope of our study. The main focus of the present study was to decipher the relative abundance of inoculant, pathogen, and the indigenous prokaryotic community composition in the tomato rhizosphere and how they link to wilting symptoms. Competitive exclusion occurring between *R. solanacearum* and antagonists was previously suggested, resulting in unsuccessful establishment of the pathogen (Upreti and Thomas, 2015). Here, by CLSM localization of P142 cells on tomato lateral root hair, root surface, as well as xylem vessels, we demonstrated a highly heterogeneous colonization pattern of the *gfp*-tagged P142 and thus the likelihood that direct interactions play a role is rather low. More likely is a priming of the tomato plants through the presence of the inoculants and/or the prokaryotic community shifts. Both inoculant strains drastically reduced the abundance of *R. solanacearum* B3B as revealed by CFU counts, qPCR, *fliC* PCR, and subsequent Southern blot hybridization and amplicon sequencing by about three orders of magnitude.

Genome sequencing revealed for both strains the presence of numerous genes involved in plant beneficial interaction, e.g., P142 carries the *phl* and the *phz* gene (Elsayed, unpublished). The *phl* gene coding for 2,4-diacetylphloroglucinol (2,4-DAPG), was previously reported for *in vitro* and *in vivo R. solanacearum* suppression (Ramadasappa et al., 2012; Zhou et al., 2012) and in addition to protists predation escaping (Jousset et al., 2006). The *phz* gene, encoding phenazine production, might also play a role in *R. solanacearum* control (Hariprasad et al., 2014). Recently, a selection strategy of potential antagonists based on the number of biological control and/or plant growth promoting related function per inoculant candidate was proposed by Mota et al. (2017). They have shown a positive correlation between the number of *in vitro* functions per antagonist and their effects on the pathogen. Both inoculant strains used in the present study affected the prokaryotic community composition in the rhizosphere. Indeed, we suspect that priority effects are at play, where the chronology of whoever comes first is determining the subsequent community assembly rule (Vannette and Fukami, 2014). Inoculants may further change the recruitment (or not) of other rhizosphere microbial members by the plant, e.g., through changes of the root exudate composition, as previously reported by Windisch et al. (2017).

Illumina amplicon sequencing analysis of 16S rRNA gene fragments of TC, TCR, TC-B63, TCR-B63, TC-P142, and TCR-P142 community DNA revealed numerous “dynamic taxa.” The most severe modulation of the rhizosphere prokaryotic community composition was observed for TCR compared to TC. Interestingly, two other genera, *Sphingobium* and *Niastella*, profited from the nutrient situation of the rhizosphere of the diseased plants. Most importantly, B3B was nearly suppressed under antagonist presence, as rhizospheres of TCR-B63 or TCR-P142 treatments had only 0.1% of *Ralstonia*-affiliated sequences, clearly demonstrating the strong biocontrol efficiency of both inoculants. Interestingly, the rhizosphere abundance of P142 was higher in the presence of *R. solanacearum* B3B (Figure 2B).

Pronounced and divergent responses in rhizosphere prokaryotic communities were found, with numerous and phylogenetically diverse OTUs showing either significantly increased or decreased relative abundance compared to controls (Tables 1, 2 and Supplementary Table S4). The most remarkable observation is the enrichment of Actinobacteria in inoculated treatments. Actinobacteria are recognized for their production of diverse bioactive compounds, their potential biological control activities, and plant growth promotion (Schrey and Tarkka, 2008; Joseph et al., 2012). Similar trends were recorded for the genus *Gaiella* that was previously described as member of the core microbiome of a disease-suppressive soil (Xue et al., 2015). The other striking observation was the pronounced enrichment of *Arthrobacter* only in TCR-B63 and TCR-P142. Egamberdieva et al. (2017) reported that *Arthrobacter crystallopoietes* had a plant growth promotion and protection effect on tomato plant, as it exhibited significant reduction of *Fusarium* infection while enhancing plant growth. Therefore, the similar responses of the prokaryotic community to the inoculation of B63 and P142 strongly suggest the indirect involvement of the plant itself, steering its root microbiota in a similar manner *via* the recruitment/stimulation of beneficial soil microbes.

Other dynamic OTUs from *Gemmatimonas*, *Devosia*, and *Sphingomonas* were enriched in response to B63 or P142, indicating that direct/indirect social interaction processes such as microbial facilitation may be involved in biocontrol. *Sphingomonas* is a strictly aerobic bacterium often characterized as an environmental oligotroph (Lauro et al., 2009; Jacquiod et al., 2017). Some *Sphingomonas* strains were shown to produce indole acetic acid (IAA), while others displayed phenazine degradation capabilities (Ma et al., 2012; Sukweenadhi et al., 2015). *Sphingomonas* was detected in lettuce rhizospheres (Schreiter et al., 2014) as well as in endophytic compartments of tomato plants (Khan et al., 2014). It is assumed that the majority of plant-associated *Sphingomonas* spp. can have a plant-protective effect (Innerebner et al., 2011; Sato et al., 2012). Moreover, genera such as *Luteolibacter* (Verrucomicrobiae) and *Ochrobactrum* were increased in all inoculated treatments. Nunes da Rocha et al. (2013) reported a rhizocompetence potential for members of *Luteolibacter* (Verrucomicrobiae) that might explain its increase. Potential antagonism of *Ochrobactrum* against phytopathogens was also reported through affecting the quorum sensing regulating the pathogen virulence factors (Czajkowski et al., 2011). However, it seems that the inoculation of antagonists tends to engineer the prokaryotic community toward enriching other beneficial bacteria. In contrast, OTUs from 20 genera were significantly decreased in TCR, mostly due to the dominance of B3B compared to TC.

The inoculation of B63 and P142 resulted in a complex response of the tomato rhizosphere bacterial communities as revealed by amplicon sequencing analysis, although it was far more pronounced for B63. This was also reflected on alpha-diversity, with an increased richness and evenness for TC-B63, indicating that it might be an important keystone species impacting the whole community *via* facilitation processes (Supplementary Table S3). Whether this effect was direct through social interactions with other species, or indirect *via* stimulation of the plant (e.g., rhizodeposition, plant defense molecules) has yet to be clarified.

## Conclusion

Control of bacterial wilt disease caused by *R. solanacearum* is an important challenge. Many strategies were proposed for controlling bacterial wilt disease. Among them, manipulating soil suppressiveness through organic amendments and managing soil suppressiveness *via* inoculant strains are considered the most promising and environmentally-friendly alternatives. Our results showed that the strains, *B. velezensis* B63 and *P. brassicacearum* P142, are promising candidates for future biocontrol of *R. solanacearum* under field conditions, through significantly lowered *R. solanacearum* densities in tomato shoots and in the rhizosphere. Amplicon sequencing revealed many dynamic taxa, likely indicating complex interactions between the inoculant strains, B3B, the prokaryotic community in the tomato rhizosphere and the plant itself. The inoculation with B63 or P142 significantly promoted specific taxa, with potential plant protection and/or growth promotion-related traits, respectively, which might, in turn, affect soil suppressiveness and increase plant defense. For the first time, 16S rRNA gene amplicon sequencing was used to demonstrate *R. solanacearum* reduction through inoculation of *in vitro* antagonists which were correlated to the reduction of wilting symptoms. Combination between cultivation-dependent and independent methods correlated well and in particular Illumina sequencing of 16S rRNA gene fragments amplified from total community DNA allowed deeper insights into the complex interaction that might lead to pathogen suppression. Present research with focus on the plant strongly points to an induction of plant systemic resistance. In summary, this study revealed that both antagonists were efficient in controlling bacterial wilt disease, but likely shifts in the rhizosphere microbiota and the antagonists contributed to the efficient control of bacterial wilt.

## Data Availability Statement

The datasets generated for this study can be found in the Sequence Read Archive (SRA), accession: PRJNA574588.

## Author Contributions

TE contributed to experimental work, data analysis, and manuscript writing. SJ contributed to data analysis and manuscript writing. EN contributed to experimental work, data analysis, and manuscript editing. SS contributed to study design and manuscript editing. KS contributed to study design and manuscript writing.

## Conflict of Interest

The authors declare that the research was conducted in the absence of any commercial or financial relationships that could be construed as a potential conflict of interest.

## Footnotes

1 https://github.com/maasha/qiime_pipe

2 https://www.ncbi.nlm.nih.gov/sra

3 https://www.ncbi.nlm.nih.gov/bioproject/PRJNA574588
