# Supplementary material for: Biocontrol of Bacterial Wilt Disease Through Complex Interaction Between Tomato Plant, Antagonists, the Indigenous Rhizosphere Microbiota, and Ralstonia solanacearum
Source: Front Microbiol. 2020 Jan 10;10:2835. doi: 10.3389/fmicb.2019.02835 (PMC6967407; doi:10.3389/fmicb.2019.02835)
Supplement: Supplementary file 1 [file Data_Sheet_1.docx]

**Supporting information file**

**_**

**Elsayed and Jacquiod et al. 2019**

**_**

**Biocontrol of bacterial wilt disease through complex interaction between tomato plant, antagonists, the indigenous rhizosphere microbiota and *Ralstonia solanacearum***

This file contains the supporting figures and tables referred in the original manuscript from Elsayed and Jacquiod et al. 2019


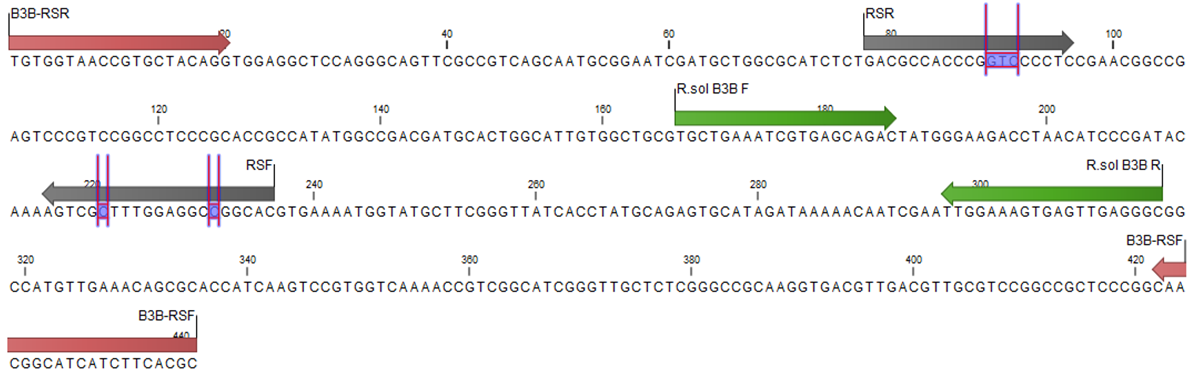


**Figure S1:**R.sol B3B F and R, the primers with an improved specificity for *R. solanacearum* (B3B, biovar 2, race 3) UDP-3-O-acyl-GlcNAc deacetylase gene. The primers RSF and RSR with mismatches (marked by vertical rectangles). The primer pair B3B-RSF and R were used to amplify a fragment of 441 bp that was subsequently cloned in *E.coli* to construct the real-time PCR standard.


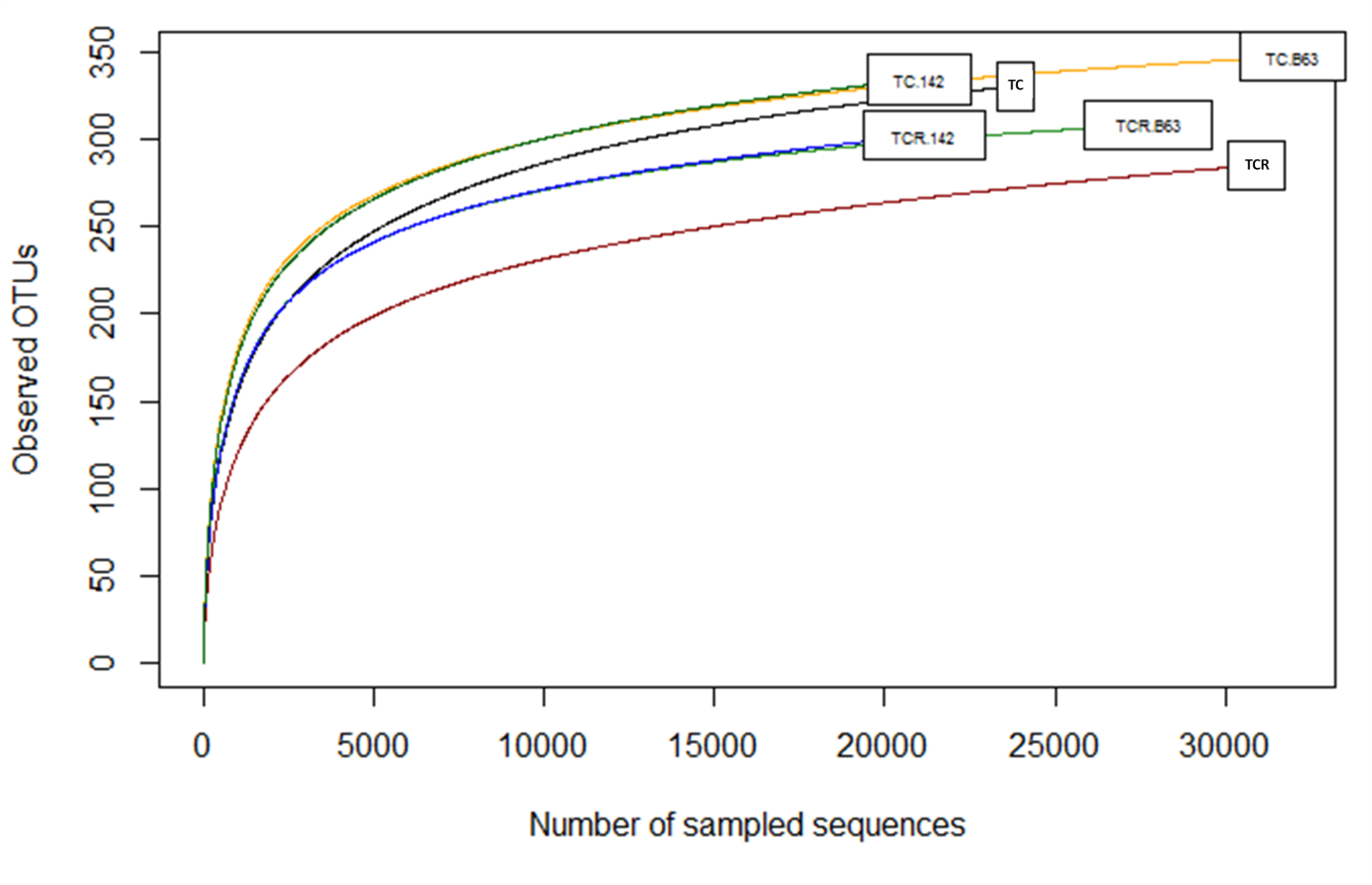


**Figure S2:** Rarefaction curves indicating the observed number of operational taxonomic units (OTUs) in prokaryotic communities relative to the number of recruited sequences, each curve is an average of the reads of four replicates.


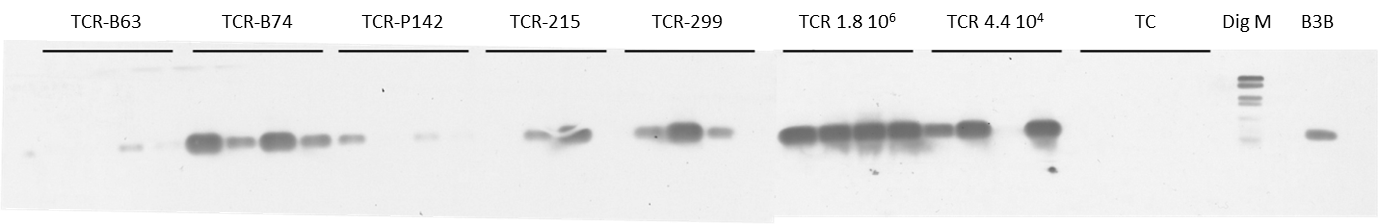


**Figure S3:** PCR-Southern blot hybridization of *fliC* gene specific for *R. solanacearum* in tomato rhizosphere total community-DNA samples, two weeks after transplanting into *R. solanacearum* infested soil. TCR 1.8 10^6^ the high *R. solanacearum* dose; TCR 4.4 10^4^, the low *R.  solanacearum* dose; TC, non-infested soil (control)

| **Target gene** | **Primers used** | **Sequence**  **5´ to 3´** | **Annealing temp.**  **(°C)** | **Reference bacteria** | **Reference** |
| --- | --- | --- | --- | --- | --- |
| BOX A1 | BOXA1R | CTACGGCAAGGCGACGCTGACG | 53 | *P. fluorescens* CHA0 | (Rademaker and De Bruijn, 1997) |
| 16S rRNA | U8-27 | AGA GTT TGA TC (AC) TGG CTC AG | 56 | *P. fluorescens* CHA0 |  |
|  |  |  |  |  |  |
|  | R1494-1514 | CTA CGG (T/C) TAC CTT GTT ACG AC |  |  |  |
| 16S rRNA | F984GC | CGCCCGGGGCGCGCCCCGGGCGGGGCGGGGGCACGGGGGGAACGCGAAGAACCTTAC | 53 | *E. coli* | (Heuer et al. 1997) |
|  | R1378-1401 | CGGTGTGTACAAGGCCCGGGAACG |  |  |  |
| *gfp* | gfp-f | AGCGTTCAACTAGCAGACCA | 60 | *gfp*-tagged P142 | (Yankson and Steck, 2009) |
|  | gfp-r | AAAGGGCAGATTGTGTGGAC |  |  |  |
| UDP-3-O-acyl-GlcNAc deacetylase | R.sol B3B F | TGCTGAAATCGTGAGCAGAC | 62 | *R. solanacearum* B3B | This study |
|  | R.sol B3B R | GCCCTCAACTCACTTTCCAA |  |  |  |
| UDP-3-O-acyl-GlcNAc  deacetylase | B3B-RSF | GCGTGAAGATGATGCCGTTG | 54 | *R. solanacearum* B3B | This study |
|  | B3B-RSR | TGTGGTAACCGTGCTACAGG |  |  |  |
| 16S rRNA | 341F | CCTAYGGGRBGCASCAG | 56 | *E. coli* | Barraud et al. (2010) |
|  | 806R | GGACTACHVGGGTWTCTAAT |  |  |  |
| *fliC* | Rsol_*fliC* _F | GAACGCCAACGGTGCGAACT | 63 | *R. solanacearum* B3B | Schönfeld et al. (2003) |
|  | Rsol_*fliC* _R | GGCGGCCTTCAGGGAGGTC |  |  |  |
| 16S rRNA | Bact1369F | CGGTGAATACGTTCYCGG | 56 | *E. coli* | Suzuki et al. (2000). |
|  | Prok1492R | GGWTACCTTGTTACGACTT |  |  |  |
|  | TM1389F (TaqMan-Probe) | CTTGTACACACCGCCCGTC |  |  |  |
|  |  |  |  |  |  |
|  |  |  |  |  |  |
| 16S rRNA | 341F | CCTAYGGGRBGCASCAG |  |  | (Jacquiod et al. 2017) |
|  | 806R | GGACTACNNGGGTATCTAAT |  |  |  |

Table S1: Primer systems, PCR conditions and reference strains used as positive controls

| **Code** | Source of rhizosphere DNA | **Replicate** | **Sequences** |
| --- | --- | --- | --- |
| TC-1 | Tomato plants non-inoculated grown in non-infested soil (healthy control; TC) | 1 | 24395 |
| TC-2 |  | 2 | 19236 |
| TC-3 |  | 3 | 15303 |
| TC-4 |  | 4 | 37016 |
| TCR-1 | Tomato plants non-inoculated grown in B3B infested soil (pathogen control; TCR) | 1 | 37458 |
| TCR-2 |  | 2 | 30359 |
| TCR-3 |  | 3 | 23683 |
| TCR-4 |  | 4 | 32675 |
| TCR-B63-1 | Tomato plants inoculated with B63 grown in B3B infested soil (TCR-B63) | 1 | 36714 |
| TCR-B63-2 |  | 2 | 26975 |
| TCR-B63-3 |  | 3 | 23238 |
| TCR-B63-4 |  | 4 | 24536 |
| TC-B63-1 | Tomato plants inoculated with B63 grown in non-infested soil (TC-63) | 1 | 29038 |
| TC-B63-2 |  | 2 | 38854 |
| TC-B63-3 |  | 3 | 35139 |
| TC-B63-4 |  | 4 | 25421 |
| TCR-P142-1 | Tomato plants inoculated with P142 grown in B3B infested soil (TCR-P142) | 1 | 22953 |
| TCR-P142-2 |  | 2 | 27600 |
| TCR-P142-3 |  | 3 | 12465 |
| TCR-P142-4 |  | 4 | 22281 |
| TC-P142-1 | Tomato plants inoculated with P142 grown in non-infested soil (TC-P142) | 1 | 30737 |
| TC-P142-2 |  | 2 | 29462 |
| TC-P142-3 |  | 3 | 4461 |
| TC-P142-4 |  | 4 | 20023 |

**Table S2:** List of samples used to generate 16S rRNA gene amplicon profiles via Illumina sequencing.

| **Alpha-diversity** | **TC** | **TCR** | **TCR-B63** | **TC-B63** | **TCR-P142** | **TC-P142** |
| --- | --- | --- | --- | --- | --- | --- |
| Richness | 297±10 bc | 238±11 a | 287±4 b | 316±5 c | 296±2 bc | 315±3 bc |
| Chao-1 | 360±3 b | 279±13 a | 338±12 b | 354±5 b | 360±5 b | 354±6 b |
| Simpson | 0.97±0 b | 0.84±0.05 a | 0.97±0 b | 0.98±0 b | 0.96±0 b | 0.98±0 b |
| Shannon | 4.29±0.08 b | 3.18±0.14 a | 4.32±0.04 bc | 4.59±0.01 c | 4.23±0.05 b | 4.54±0.03 bc |
| Equitability | 0.75±0.01 b | 0.58±0.03 a | 0.76±0.01 b | 0.8±0 b | 0.74±0.01 b | 0.79±0.01 b |

**Table S3:** Alpha-diversity analysis of rhizospheric bacterial communities at OTUs level (average ±Standard error of the mean). The total diversity was assessed through estimation of the richness and Chao-1, while the community evenness was assessed by means of Simpson, Shannon and Shannon’s Equitabily indices. Statistically significant trends were inferred using a multi-group GLM ANOVA corrected with a post-hoc Tukey LSD test (*p*< 0.05). Treatments sharing the same letters are non-significantly different across each line. Highlighted in green are significantly highest recorded values, significantly lowest values are highlighted in red.

| ***Class*** | ***Genus*** | **OTUs** | TC | TCR | TC-B63 | TCR-B63 | TC-P142 | TCR-P142 |
| --- | --- | --- | --- | --- | --- | --- | --- | --- |
| *Actinobacteria* | *Aciditerrimonas* | OTU_574 | 0.1 ± 0^b^ | 0.1 ± 0^a^ | **0.6 ± 0.1^d^** | **0.3 ± 0^c^** | **0.3 ± 0.1^c^** | 0.2 ± 0^bc^ |
|  | *Acidothermus* | OTU_815 | 0 ± 0^ab^ | 0 ± 0^a^ | **0.1 ± 0^c^** | **0.1 ± 0^c^** | 0 ± 0^b^ | 0 ± 0^ab^ |
|  | *Aeromicrobium* | OTU_526 | 0.1 ± 0^b^ | 0 ± 0^a^ | **0.5 ± 0.1^d^** | **0.4 ± 0.1^cd^** | **0.3 ± 0.1^c^** | **0.3 ± 0.1^c^** |
|  | *Arthrobacter* | OTU_37 | 4.2 ± 1.3^b^ | 1 ± 0.7^a^ | 6.5 ± 1.3^bc^ | **9 ± 1.1^c^** | 6.2 ± 2.9^bc^ | **11 ± 1.3^c^** |
|  |  | OTU_102 | 0.3 ± 0.1^b^ | 0.1 ± 0^a^ | 0.1 ± 0^a^ | 0.1 ± 0^a^ | 0.1 ± 0^a^ | 0.1 ± 0^ab^ |
|  | *Blastococcus* | OTU_1218 | 0.1 ± 0^ab^ | 0 ± 0^a^ | **0.2 ± 0.1^c^** | 0.1 ± 0^bc^ | 0.1 ± 0.1^bc^ | 0.1 ± 0^b^ |
|  | *Cellulomonas* | OTU_172 | 0.1 ± 0^b^ | 0.1 ± 0^a^ | **0.2 ± 0^cd^** | **0.3 ± 0.1^e^** | 0.2 ± 0.1^bc^ | **0.3 ± 0^de^** |
|  | *Conexibacter* | OTU_1362 | 0.2 ± 0^b^ | 0.1 ± 0^a^ | **0.6 ± 0.1^c^** | **0.7 ± 0.1^c^** | 0.3 ± 0.1^b^ | 0.2 ± 0.1^b^ |
|  | *Corynebacterium* | OTU_2093 | 0.2 ± 0^b^ | 0 ± 0^a^ | 0.1 ± 0^a^ | 0.1 ± 0^ab^ | 0.1 ± 0.1^ab^ | 0.2 ± 0^b^ |
|  | *Curtobacterium* | OTU_45 | 0.7 ± 0.2^b^ | 0.2 ± 0.1^a^ | 0.5 ± 0.2^b^ | 0.6 ± 0.2^b^ | 0.6 ± 0.2^b^ | 0.7 ± 0.1^b^ |
|  | *Gaiella* | OTU_466 | 1.1 ± 0.2^b^ | 0.8 ± 0.2^a^ | **2.9 ± 0.3^d^** | **2.2 ± 0.3^cd^** | **1.8 ± 0.4^c^** | **1.6 ± 0.2^c^** |
|  | *Iamia* | OTU_1497 | 0 ± 0^a^ | 0 ± 0^a^ | **0.1 ± 0^c^** | **0.1 ± 0^b^** | **0.1 ± 0^b^** | **0.1 ± 0^b^** |
|  | *Ilumatobacter* | OTU_547 | 0.1 ± 0^a^ | 0.1 ± 0^a^ | **0.3 ± 0.1^d^** | **0.1 ± 0^b^** | **0.2 ± 0^c^** | **0.2 ± 0^bc^** |
|  | *Kocuria* | OTU_290 | 0.4 ± 0.1^bc^ | 0.1 ± 0.1^a^ | 0.3 ± 0.1^ab^ | 0.9 ± 0.5^cd^ | 0.4 ± 0.2^bc^ | **1.4 ± 0.7^d^** |
|  | *Kribbella* | OTU_1822 | 0 ± 0^ab^ | 0 ± 0^a^ | **0.1 ± 0^c^** | **0.1 ± 0^c^** | 0 ± 0^ab^ | 0 ± 0^b^ |
|  | *Microbacterium* | OTU_1817 | 0 ± 0^a^ | 0 ± 0^a^ | **0.1 ± 0^b^** | **0.1 ± 0.1^b^** | 0 ± 0^a^ | 0.1 ± 0.1^ab^ |
|  | *Mycobacterium* | OTU_1971 | 0.2 ± 0.1^c^ | 0.1 ± 0^b^ | 0 ± 0^a^ | 0 ± 0^a^ | 0.2 ± 0.1^bc^ | 0.1 ± 0^bc^ |
|  | *Nocardioides* | OTU_461 | 0.2 ± 0^bc^ | 0.1 ± 0.1^ac^ | 0.1 ± 0^a^ | 0.1 ± 0^ab^ | 0.2 ± 0.1^ac^ | 0.3 ± 0.1^c^ |
|  |  | OTU_1867 | 0 ± 0^ab^ | 0 ± 0^a^ | **0.1 ± 0^c^** | 0.1 ± 0^bc^ | 0.1 ± 0.1^bc^ | 0.1 ± 0^bc^ |
|  |  | OTU_1493 | 0 ± 0^ab^ | 0 ± 0^a^ | **0.1 ± 0^c^** | 0.1 ± 0^bc^ | 0.1 ± 0^bc^ | 0 ± 0^b^ |
|  |  | OTU_371 | 0 ± 0^a^ | 0 ± 0^a^ | **0.6 ± 0.2^b^** | **0.7 ± 0.1^b^** | 0.1 ± 0^a^ | 0.1 ± 0^a^ |
|  |  | OTU_1542 | 0 ± 0^a^ | 0 ± 0^a^ | **0.2 ± 0^c^** | **0.2 ± 0^bc^** | **0.2 ± 0.1^bc^** | **0.1 ± 0^b^** |
|  | *Ornithinicoccus* | OTU_1838 | 0 ± 0^ab^ | 0 ± 0^a^ | **0.1 ± 0^c^** | **0.1 ± 0^c^** | 0 ± 0^ab^ | 0 ± 0^bc^ |
|  | *Phycicoccus* | OTU_243 | 0.1 ± 0^ab^ | 0 ± 0^a^ | **0.2 ± 0^c^** | 0.1 ± 0^bc^ | 0.1 ± 0.1^ac^ | 0.1 ± 0^bc^ |
|  | *Rhodococcus* | OTU_2103 | 0 ± 0^ac^ | 0 ± 0^a^ | 0.1 ± 0^bcd^ | **0.1 ± 0.1^d^** | 0 ± 0^ab^ | 0.1 ± 0^cd^ |
|  |  | OTU_1762 | 0 ± 0^ab^ | 0 ± 0^a^ | 0.1 ± 0^bc^ | **0.1 ± 0^c^** | 0 ± 0^ab^ | **0.1 ± 0^c^** |
|  | *Rubrobacter* | OTU_0 | 3.1 ± 1.8^bc^ | 0.5 ± 0.6^a^ | 5.2 ± 1.5^c^ | 1.3 ± 0.8^ab^ | 7.7 ± 3.3^c^ | 2.8 ± 0.7^bc^ |
|  | *Salinibacterium* | OTU_1281 | 0.7 ± 0.3^b^ | 0.2 ± 0.1^a^ | 0.6 ± 0.2^b^ | 0.8 ± 0.1^b^ | 0.7 ± 0.2^b^ | 0.8 ± 0.2^b^ |
|  | *Solirubrobacter* | OTU_1591 | 0 ± 0^ab^ | 0 ± 0^a^ | **0.1 ± 0^d^** | **0.1 ± 0^cd^** | 0.1 ± 0^bc^ | 0 ± 0^ac^ |
|  | *Streptomyces* | OTU_721 | 0.5 ± 0.3^bc^ | 0.2 ± 0.1^a^ | 0.6 ± 0.2^bc^ | 1.1 ± 0.3^c^ | 0.3 ± 0.1^ab^ | 0.7 ± 0.2^c^ |
|  |  | OTU_216 | 0.3 ± 0.1^bc^ | 0.1 ± 0^a^ | 0.3 ± 0^bc^ | 0.4 ± 0.1^c^ | 0.2 ± 0.1^ab^ | 0.2 ± 0.1^ab^ |
|  | *Terrabacter* | OTU_385 | 0.1 ± 0^ab^ | 0.1 ± 0^a^ | **0.4 ± 0.1^c^** | 0.3 ± 0.1^bc^ | 0.3 ± 0.2^bc^ | **0.4 ± 0.1^c^** |
|  | *Unclass_Acidimicrobiales* | OTU_488 | 0 ± 0^a^ | 0 ± 0^a^ | **0.1 ± 0^bc^** | **0.1 ± 0^c^** | **0.1 ± 0^bc^** | 0 ± 0^ab^ |
| *Alphaproteobacteria* | *Acidisphaera* | OTU_1216 | 0.2 ± 0^b^ | 0.2 ± 0.1^ab^ | 0.1 ± 0^a^ | 0.1 ± 0^a^ | 0.2 ± 0^b^ | 0.2 ± 0.1^b^ |
|  | *Asticcacaulis* | OTU_12 | 3.1 ± 0.5^b^ | 5.5 ± 5.2^b^ | 0.7 ± 0.5^a^ | 0.8 ± 0.2^a^ | 2 ± 1.4^ab^ | 1.7 ± 0.6^ab^ |
|  | *Azospirillum* | OTU_427 | 0.1 ± 0^c^ | 0.1 ± 0.1^ac^ | 0.1 ± 0.1^bc^ | 0 ± 0^ab^ | 0.1 ± 0^bc^ | 0 ± 0^a^ |
|  | *Bauldia* | OTU_1342 | 0.1 ± 0^a^ | 0.1 ± 0^a^ | **0.2 ± 0^b^** | **0.2 ± 0^b^** | **0.2 ± 0.1^b^** | **0.2 ± 0.1^b^** |
|  | *Bosea* | OTU_291 | 0.1 ± 0^a^ | 0.1 ± 0.1^a^ | 0.3 ± 0.1^a^ | **0.6 ± 0.3^b^** | 0.2 ± 0^a^ | 0.2 ± 0^a^ |
|  | *Bradyrhizobium* | OTU_10 | 2.1 ± 0.6^bc^ | 1 ± 0.3^a^ | 2.3 ± 0.4^c^ | 2.4 ± 0.2^c^ | 1.7 ± 0.6^bc^ | 1.4 ± 0.2^ab^ |
|  | *Brevundimonas* | OTU_590 | 0.2 ± 0.1^ab^ | 0.2 ± 0.1^a^ | 0.4 ± 0.1^bc^ | **0.5 ± 0.1^c^** | **0.8 ± 0.4^c^** | **0.6 ± 0.1^c^** |
|  | *Devosia* | OTU_255 | 1.5 ± 0.2^c^ | 0.9 ± 0.1^b^ | 0.7 ± 0.1^b^ | 0.4 ± 0^a^ | 1.5 ± 0.2^c^ | 1.2 ± 0.3^c^ |
|  |  | OTU_93 | 1.5 ± 0.2^ab^ | 1.2 ± 0.4^a^ | **2.6 ± 0.5^c^** | **2.4 ± 0.3^c^** | 2.3 ± 0.3^bc^ | 2.3 ± 0.5^bc^ |
|  |  | OTU_244 | 0.3 ± 0.1^a^ | 0.4 ± 0.2^a^ | **0.7 ± 0.3^bc^** | 0.4 ± 0.1^a^ | **1.1 ± 0.1^c^** | 0.5 ± 0^ab^ |
|  | *Dongia* | OTU_2117 | 0.3 ± 0.1^bc^ | 0.2 ± 0.1^a^ | 0.4 ± 0.1^c^ | 0.4 ± 0^c^ | 0.3 ± 0^ac^ | 0.2 ± 0.1^ab^ |
|  | *Hyphomicrobium* | OTU_1280 | 0 ± 0^ab^ | 0 ± 0^a^ | **0.1 ± 0^d^** | 0.1 ± 0^bc^ | **0.1 ± 0^cd^** | 0 ± 0^ac^ |
|  |  | OTU_2062 | 0 ± 0^a^ | 0 ± 0^a^ | **0.1 ± 0^c^** | **0 ± 0^b^** | **0.1 ± 0^bc^** | **0 ± 0^b^** |
|  | *Mesorhizobium* | OTU_124 | 0.5 ± 0.4^bd^ | 0.2 ± 0.1^a^ | 0.2 ± 0.1^ab^ | 0.3 ± 0^abc^ | 0.6 ± 0.2^d^ | 0.5 ± 0.1^cd^ |
|  | *Methylocapsa* | OTU_268 | 0.2 ± 0.1^b^ | 0.1 ± 0^a^ | 0.1 ± 0^a^ | 0.1 ± 0^ab^ | 0.1 ± 0^ab^ | 0.1 ± 0^a^ |
|  | *Microvirga* | OTU_377 | 0.2 ± 0.1^b^ | 0.1 ± 0^a^ | **0.5 ± 0.1^c^** | 0.2 ± 0.1^b^ | **0.4 ± 0.1^c^** | 0.3 ± 0^bc^ |
|  |  | OTU_1448 | 0 ± 0^ab^ | 0 ± 0^a^ | **0.2 ± 0.1^c^** | 0.1 ± 0^bc^ | **0.1 ± 0.1^c^** | 0.1 ± 0^bc^ |
|  | *Novosphingobium* | OTU_56 | 0.5 ± 0.7^b^ | 0.1 ± 0^a^ | 0.1 ± 0^ab^ | 0.1 ± 0^a^ | 0.2 ± 0^ab^ | 0.1 ± 0^ab^ |
|  |  | OTU_1162 | 0.2 ± 0.1^b^ | 0.1 ± 0^a^ | 0.3 ± 0^b^ | 0.2 ± 0.1^b^ | 0.3 ± 0.1^b^ | 0.2 ± 0.1^b^ |
|  | *Ochrobactrum* | OTU_669 | 0.3 ± 0.1^a^ | 0.3 ± 0.1^a^ | **0.7 ± 0.1^b^** | **0.8 ± 0^bc^** | **0.9 ± 0.2^bc^** | **1 ± 0.2^c^** |
|  | *Pedomicrobium* | OTU_714 | 0 ± 0^ab^ | 0 ± 0^a^ | **0.1 ± 0^c^** | 0 ± 0^ab^ | 0.1 ± 0.1^bc^ | 0 ± 0^ab^ |
|  |  | OTU_420 | 0 ± 0^a^ | 0 ± 0^a^ | **0.1 ± 0^b^** | 0 ± 0^a^ | **0.1 ± 0^b^** | 0 ± 0^a^ |
|  | *Phyllobacterium* | OTU_1357 | 0.1 ± 0.1^ab^ | 0 ± 0^a^ | 0.2 ± 0^bc^ | 0.1 ± 0^bc^ | **0.2 ± 0.1^c^** | **0.2 ± 0.1^c^** |
|  | *Porphyrobacter* | OTU_1610 | 0.2 ± 0.1^b^ | 0.1 ± 0.1^ab^ | 0.1 ± 0^ab^ | 0.1 ± 0^a^ | 0.2 ± 0.1^b^ | 0.2 ± 0.1^ab^ |
|  | *Pseudolabrys* | OTU_118 | 0.3 ± 0^ab^ | 0.2 ± 0.1^a^ | **0.8 ± 0.1^c^** | **0.8 ± 0.1^c^** | 0.4 ± 0.2^b^ | 0.4 ± 0.1^b^ |
|  |  | OTU_1424 | 0.3 ± 0.2^b^ | 0.2 ± 0^a^ | **0.9 ± 0.1^d^** | **0.6 ± 0.1^cd^** | 0.4 ± 0.1^bc^ | 0.4 ± 0.1^b^ |
|  | *Rhizobium* | OTU_173 | 2 ± 0.7^c^ | 1.1 ± 0.5^bc^ | 0.3 ± 0.1^a^ | 1.1 ± 0.4^bc^ | 0.6 ± 0.2^ab^ | 0.8 ± 0.2^b^ |
|  | *Rhodoligotrophos* | OTU_428 | 0.1 ± 0^ab^ | 0 ± 0^a^ | **0.4 ± 0.1^d^** | **0.1 ± 0.1^c^** | **0.3 ± 0.1^d^** | 0.1 ± 0^bc^ |
|  | *Rhodoplanes* | OTU_1371 | 0.5 ± 0.1^b^ | 0.3 ± 0.1^a^ | **0.8 ± 0.2^d^** | **0.7 ± 0.1^cd^** | 0.6 ± 0.1^bd^ | 0.5 ± 0^bc^ |
|  | *Roseomonas* | OTU_1948 | 0.2 ± 0^c^ | 0.1 ± 0^bc^ | 0 ± 0^a^ | 0 ± 0^a^ | 0.1 ± 0^bc^ | 0.1 ± 0^ab^ |
|  | *Skermanella* | OTU_185 | 0 ± 0^a^ | 0 ± 0^a^ | **0.1 ± 0^b^** | **0.1 ± 0^b^** | **0.1 ± 0^b^** | **0.1 ± 0^b^** |
|  | *Sphingobium* | OTU_18 | 3.6 ± 1.3^ab^ | 2.1 ± 0.7^a^ | 5.2 ± 0.9^bc^ | **7.2 ± 1.8^cd^** | 5.1 ± 0.4^bc^ | **11.1 ± 3.1^d^** |
|  |  | OTU_1976 | 0.2 ± 0.2^a^ | **0.9 ± 0.9^b^** | 0.2 ± 0^ab^ | 0.3 ± 0.1^ab^ | 0.2 ± 0.1^a^ | 0.4 ± 0.1^ab^ |
|  |  | OTU_350 | 0.1 ± 0.1^a^ | 0 ± 0.1^a^ | 0 ± 0.1^a^ | 0 ± 0^a^ | 0 ± 0^a^ | 0 ± 0^a^ |
|  |  | OTU_417 | 0 ± 0^a^ | 0 ± 0^a^ | **0.3 ± 0.1^c^** | **0.2 ± 0^b^** | 0.1 ± 0^a^ | 0 ± 0^a^ |
|  |  | OTU_25 | 0 ± 0^a^ | 0 ± 0^a^ | **0.1 ± 0^b^** | **0.1 ± 0^b^** | **0.1 ± 0^b^** | **0.1 ± 0^b^** |
|  | *Sphingomonas* | OTU_107 | 1.2 ± 0.2^b^ | 0.5 ± 0.1^a^ | **2.5 ± 0.6^c^** | **2.4 ± 0.2^c^** | 1.6 ± 0.5^b^ | 1.5 ± 0.3^b^ |
|  |  | OTU_2099 | 1.1 ± 0.3^b^ | 0.4 ± 0.1^a^ | 1.4 ± 0.3^bc^ | **2 ± 0.2^c^** | 0.9 ± 0.1^b^ | 1.3 ± 0^bc^ |
|  |  | OTU_33 | 1 ± 0.1^b^ | 0.4 ± 0.2^a^ | **2.3 ± 0.2^d^** | 1.3 ± 0.2^bc^ | **2.2 ± 0.4^cd^** | 1.4 ± 0.2^bc^ |
|  |  | OTU_62 | 0.1 ± 0^b^ | 0.1 ± 0^a^ | **0.4 ± 0^d^** | 0.2 ± 0^bc^ | **0.4 ± 0^d^** | **0.3 ± 0^cd^** |
|  |  | OTU_1494 | 0 ± 0^ab^ | 0 ± 0^a^ | **0.1 ± 0^e^** | 0 ± 0^bc^ | **0.1 ± 0^de^** | **0.1 ± 0^cd^** |
|  |  | OTU_576 | 0 ± 0^a^ | 0 ± 0^a^ | **0.1 ± 0^b^** | **0.2 ± 0.1^c^** | 0 ± 0^a^ | 0 ± 0^a^ |
|  | *Unclass_Erythrobacteraceae* | OTU_1653 | 0.2 ± 0.1^b^ | 0.1 ± 0^a^ | **0.3 ± 0^c^** | 0.1 ± 0^ab^ | **0.3 ± 0^c^** | 0.2 ± 0.1^bc^ |
|  | *Unclass_Rhizobiales* | OTU_29 | 0.2 ± 0.1^bd^ | 0.1 ± 0^a^ | 0.3 ± 0^d^ | 0.1 ± 0^ab^ | 0.2 ± 0.1^cd^ | 0.1 ± 0^abc^ |
|  | *Unclass_Rhodospirillaceae* | OTU_108 | 0.4 ± 0.1^b^ | 0.1 ± 0.1^a^ | **1.6 ± 0.3^c^** | **2.1 ± 0.2^c^** | 0.3 ± 0.1^b^ | 0.3 ± 0^b^ |
|  | *Unclass_Sphingomonadaceae* | OTU_556 | 0.1 ± 0.1^b^ | 0 ± 0^a^ | **0.3 ± 0.1^d^** | 0.1 ± 0^bc^ | **0.2 ± 0^cd^** | 0.1 ± 0^b^ |
| *Betaproteobacteria* | *Acidovorax* | OTU_296 | 0.2 ± 0.1^a^ | 0.2 ± 0.2^a^ | **0.8 ± 0.1^b^** | **0.8 ± 0.1^b^** | **1 ± 1^b^** | 0.5 ± 0.2^ab^ |
|  | *Albidiferax* | OTU_699 | 0.1 ± 0^c^ | 0 ± 0^bc^ | 0 ± 0^a^ | 0 ± 0^ab^ | 0.1 ± 0^c^ | 0 ± 0^ac^ |
|  | *Aquabacterium* | OTU_813 | 0 ± 0^ab^ | 0 ± 0^a^ | **0.2 ± 0^d^** | 0.1 ± 0^bc^ | **0.1 ± 0.1^c^** | 0.1 ± 0^bc^ |
|  | *Burkholderia* | OTU_2109 | 0.6 ± 0.1^b^ | 0.2 ± 0.1^a^ | 0.3 ± 0.1^ab^ | 0.6 ± 0.2^b^ | 0.2 ± 0.1^a^ | 0.3 ± 0.1^ab^ |
|  |  | OTU_992 | 0.3 ± 0.2^c^ | 0.1 ± 0^ab^ | 0 ± 0^a^ | 0 ± 0^a^ | 0.1 ± 0.1^ab^ | 0.1 ± 0^b^ |
|  |  | OTU_357 | 0.1 ± 0^d^ | 0.1 ± 0^ac^ | 0 ± 0^a^ | 0 ± 0^ab^ | 0.1 ± 0^bc^ | 0.1 ± 0^cd^ |
|  |  | OTU_1818 | 0.1 ± 0^b^ | 0 ± 0^a^ | 0 ± 0^a^ | 0 ± 0^a^ | 0 ± 0^a^ | 0 ± 0^a^ |
|  | *Collimonas* | OTU_657 | 0.1 ± 0^a^ | 0 ± 0^a^ | **0.1 ± 0^b^** | 0 ± 0^a^ | **0.1 ± 0^b^** | 0.1 ± 0^ab^ |
|  | *Cupriavidus* | OTU_2080 | 0 ± 0^a^ | 0 ± 0^a^ | 0 ± 0^ab^ | **0 ± 0^bc^** | 0 ± 0^a^ | **0.1 ± 0^c^** |
|  | *Curvibacter* | OTU_370 | 0.4 ± 0.1^c^ | 0.2 ± 0.1^bc^ | 0 ± 0^a^ | 0 ± 0^a^ | 0.2 ± 0.1^bc^ | 0.2 ± 0.1^b^ |
|  | *Hydrogenophaga* | OTU_1403 | 0 ± 0^a^ | 0 ± 0^a^ | **0.1 ± 0^b^** | 0 ± 0^a^ | 0 ± 0^ab^ | 0 ± 0^ab^ |
|  | *Ideonella* | OTU_529 | 0.1 ± 0^a^ | 0.1 ± 0^a^ | **0.5 ± 0.2^c^** | 0.2 ± 0.1^ab^ | **0.4 ± 0.2^bc^** | 0.2 ± 0.1^a^ |
|  | *Massilia* | OTU_100 | 4 ± 2.1^b^ | 3.5 ± 4.3^ab^ | 1 ± 0.1^a^ | 2.4 ± 1^ab^ | 1.2 ± 0.5^ab^ | 2.9 ± 1.6^ab^ |
|  |  | OTU_742 | 0 ± 0^a^ | 0 ± 0^a^ | 0.1 ± 0^ab^ | **0.1 ± 0^b^** | 0.1 ± 0^ab^ | **0.1 ± 0^b^** |
|  | *Nitrosospira* | OTU_403 | 0.4 ± 0.2^d^ | 0.1 ± 0^ab^ | 0.3 ± 0.1^cd^ | 0.3 ± 0.1^cd^ | 0.2 ± 0.2^bc^ | 0 ± 0^a^ |
|  |  | OTU_2006 | 0 ± 0^a^ | 0 ± 0^a^ | **0.1 ± 0^b^** | 0 ± 0^a^ | 0 ± 0^a^ | 0 ± 0^a^ |
|  | *Ralstonia* | OTU_1 | 0 ± 0^a^ | **35.8 ± 15.7^b^** | 0 ± 0^a^ | 0.1 ± 0.1^a^ | 0 ± 0^a^ | 0.1 ± 0.2^a^ |
|  |  | OTU_115 | 0 ± 0^a^ | **0.3 ± 0.1^b^** | 0 ± 0^a^ | 0 ± 0^a^ | 0 ± 0^a^ | 0 ± 0^a^ |
|  |  | OTU_116 | 0 ± 0^a^ | **0.2 ± 0.1^b^** | 0 ± 0^a^ | 0 ± 0^a^ | 0 ± 0^a^ | 0 ± 0^a^ |
|  | *Ramlibacter* | OTU_1253 | 0 ± 0^a^ | 0 ± 0^ab^ | **0.1 ± 0^b^** | 0 ± 0^ab^ | 0.1 ± 0^ab^ | 0 ± 0^ab^ |
|  | *Shinella* | OTU_16 | 5.1 ± 1.7^b^ | 4.1 ± 0.5^b^ | 2.5 ± 0.5^a^ | **8.6 ± 1.6^c^** | 2.4 ± 0.4^a^ | 4.7 ± 0.9^b^ |
|  | *Thiobacillus* | OTU_819 | 0 ± 0^ab^ | 0 ± 0^a^ | **0.1 ± 0.1^c^** | 0.1 ± 0^b^ | 0.1 ± 0.1^b^ | 0 ± 0^ab^ |
| *Gammaproteobacteria* | *Arenimonas* | OTU_1479 | 0 ± 0^ab^ | 0 ± 0^a^ | **0.1 ± 0^d^** | 0 ± 0^bc^ | **0.1 ± 0^d^** | **0.1 ± 0^cd^** |
|  |  | OTU_1350 | 0 ± 0^a^ | 0 ± 0^a^ | **0.1 ± 0^c^** | 0 ± 0^a^ | **0.1 ± 0.1^b^** | 0 ± 0^a^ |
|  | *Dokdonella* | OTU_596 | 0.1 ± 0.1^a^ | 0 ± 0^a^ | **0.3 ± 0.1^b^** | **0.4 ± 0.1^b^** | 0 ± 0^a^ | 0 ± 0^a^ |
|  |  | OTU_1099 | 0 ± 0^ab^ | 0 ± 0^a^ | **0.1 ± 0^c^** | 0 ± 0^ab^ | 0 ± 0^bc^ | 0 ± 0^ab^ |
|  | *Dyella* | OTU_968 | 1.1 ± 0.7^b^ | 0.2 ± 0.1^a^ | 0.3 ± 0.1^a^ | 0.2 ± 0.1^a^ | 0.1 ± 0^a^ | 0.1 ± 0.1^a^ |
|  |  | OTU_1223 | 0.5 ± 0.1^b^ | 0.1 ± 0^a^ | 0 ± 0^a^ | 0.1 ± 0^a^ | 0.1 ± 0.1^a^ | 0.1 ± 0.1^a^ |
|  | *Legionella* | OTU_352 | 0 ± 0^a^ | 0 ± 0^a^ | 0 ± 0^a^ | 0 ± 0^a^ | **0.1 ± 0.1^b^** | 0 ± 0^a^ |
|  | *Lysobacter* | OTU_329 | 0.4 ± 0.2^a^ | 0.3 ± 0.2^a^ | 0.7 ± 0.2^ab^ | **1.4 ± 0.5^b^** | 0.4 ± 0.2^a^ | 0.4 ± 0.1^a^ |
|  |  | OTU_1962 | 0.1 ± 0^ab^ | 0.1 ± 0^a^ | **0.4 ± 0.1^c^** | 0.2 ± 0^b^ | **0.3 ± 0.1^c^** | 0.2 ± 0.1^b^ |
|  |  | OTU_433 | 0.1 ± 0.1^ab^ | 0 ± 0^a^ | 0.1 ± 0^ab^ | **0.3 ± 0.2^c^** | 0.1 ± 0^b^ | **0.5 ± 0.1^c^** |
|  |  | OTU_304 | 0 ± 0^ab^ | 0 ± 0^a^ | **0.1 ± 0^c^** | 0 ± 0^bc^ | **0.1 ± 0^c^** | 0 ± 0^ab^ |
|  | *Pseudoxanthomonas* | OTU_209 | 0 ± 0^ab^ | 0 ± 0^a^ | **0.2 ± 0.1^d^** | **0.1 ± 0^c^** | **0.1 ± 0^c^** | 0.1 ± 0^bc^ |
|  |  | OTU_1947 | 0 ± 0^a^ | 0 ± 0^a^ | 0.1 ± 0.1^ab^ | 0 ± 0^a^ | **0.2 ± 0.2^b^** | 0 ± 0^a^ |
|  | *Rhizobacter* | OTU_363 | 0 ± 0^a^ | 0 ± 0^a^ | **0.4 ± 0.2^c^** | **0.2 ± 0.1^b^** | **0.3 ± 0.2^bc^** | **0.1 ± 0^b^** |
|  | *Rhodanobacter* | OTU_282 | 9 ± 2.7^c^ | 2.2 ± 0.6^b^ | 0.7 ± 0^a^ | 1.2 ± 0.2^ab^ | 1.3 ± 0.6^ab^ | 1.6 ± 0.5^b^ |
|  |  | OTU_1861 | 0.1 ± 0^b^ | 0 ± 0^a^ | 0.1 ± 0^b^ | 0.1 ± 0.1^b^ | 0 ± 0^a^ | 0 ± 0^a^ |
|  | *Rudaea* | OTU_278 | 0.7 ± 0.2^bc^ | 0.5 ± 0.2^b^ | 0 ± 0^a^ | 0 ± 0^a^ | 1 ± 0.5^c^ | 0.7 ± 0.2^bc^ |
|  | *Thermomonas* | OTU_2027 | 0.1 ± 0^a^ | 0 ± 0^a^ | **0.3 ± 0^b^** | **0.2 ± 0^b^** | **0.3 ± 0.1^b^** | **0.3 ± 0^b^** |
|  | *Unclass_Enterobacteriaceae* | OTU_2015 | 3.2 ± 1.2^c^ | 0.5 ± 0.4^a^ | 3.2 ± 0.6^c^ | 1.2 ± 0.5^b^ | 4.1 ± 1.2^c^ | 1.7 ± 0.2^bc^ |
|  | *Unclass_Xanthomonadaceae* | OTU_153 | 0 ± 0^ab^ | 0 ± 0^a^ | **0.2 ± 0.1^c^** | **0.1 ± 0.1^c^** | 0.1 ± 0^bc^ | 0.1 ± 0^ac^ |
| *Deltaproteobacteria* | *Anaeromyxobacter* | OTU_2119 | 0.1 ± 0^bc^ | 0.1 ± 0^bc^ | 0 ± 0^ab^ | 0 ± 0^a^ | 0.1 ± 0^c^ | 0 ± 0^ab^ |
|  | *Bdellovibrio* | OTU_724 | 0.2 ± 0.1^c^ | 0.2 ± 0.1^bc^ | 0.1 ± 0^a^ | 0 ± 0^a^ | 0.2 ± 0^bc^ | 0.1 ± 0^ab^ |
|  | *Byssovorax* | OTU_1609 | 0 ± 0^ab^ | 0 ± 0^a^ | 0.1 ± 0^bc^ | 0 ± 0^ab^ | 0 ± 0^ac^ | **0.1 ± 0^c^** |
|  | *Cystobacter* | OTU_632 | 0 ± 0^a^ | 0 ± 0^a^ | **0.1 ± 0.1^b^** | **0.1 ± 0^b^** | 0 ± 0^a^ | 0 ± 0^a^ |
|  | *Kofleria* | OTU_170 | 0 ± 0^a^ | 0 ± 0^a^ | **0.1 ± 0.1^c^** | **0.1 ± 0^bc^** | 0 ± 0^ab^ | 0 ± 0^a^ |
| *Anaerolineae* | *Unclass_Anaerolineaceae* | OTU_2059 | 0.1 ± 0^ab^ | 0 ± 0^a^ | **0.2 ± 0^c^** | 0.1 ± 0^bc^ | 0.1 ± 0^bc^ | 0.1 ± 0^b^ |
| *Bacilli* | *Bacillus* | OTU_32 | 0.9 ± 0.5^c^ | 0 ± 0^a^ | 0.2 ± 0.1^b^ | 0.2 ± 0^b^ | 0.3 ± 0.1^b^ | 0.2 ± 0.1^b^ |
|  |  | OTU_1532 | 0.2 ± 0.1^b^ | 0 ± 0^a^ | 0.1 ± 0^ab^ | 0 ± 0^a^ | 0.1 ± 0^a^ | 0 ± 0^a^ |
|  |  | OTU_514 | 0.1 ± 0.1^b^ | 0 ± 0^a^ | 0.1 ± 0^ab^ | 0 ± 0^a^ | 0.1 ± 0^ab^ | 0 ± 0^a^ |
|  |  | OTU_55 | 0.1 ± 0.1^b^ | 0 ± 0^a^ | 0.1 ± 0^b^ | 0 ± 0^ab^ | 0 ± 0^ab^ | 0 ± 0^ab^ |
|  | *Paenibacillus* | OTU_1311 | 0.1 ± 0^ab^ | 0 ± 0^a^ | 0.1 ± 0.1^b^ | 0.1 ± 0^ab^ | **0.3 ± 0.1^c^** | 0.1 ± 0.1^ab^ |
|  |  | OTU_1611 | 0 ± 0^a^ | 0 ± 0^a^ | **0.4 ± 0.2^b^** | **0.4 ± 0^b^** | 0 ± 0^a^ | 0 ± 0^a^ |
|  | *Sporosarcina* | OTU_38 | 0.3 ± 0.2^b^ | 0 ± 0^a^ | 0.1 ± 0.1^ab^ | 0 ± 0^a^ | 0.2 ± 0.1^ab^ | 0.1 ± 0^a^ |
| *Bacteroidia* | *Bacteroides* | OTU_2029 | 0.1 ± 0.1^b^ | 0 ± 0^a^ | 0 ± 0^a^ | 0 ± 0^a^ | 0 ± 0^a^ | 0 ± 0^a^ |
|  | *Unclass_Bacteroidales* | OTU_160 | 0.1 ± 0^ab^ | 0 ± 0^a^ | **0.5 ± 0.2^d^** | **0.4 ± 0.1^cd^** | 0.2 ± 0.1^bc^ | 0.1 ± 0.1^b^ |
| *Chloroflexia* | *Roseiflexus* | OTU_1958 | 0 ± 0^ab^ | 0 ± 0^a^ | **0.3 ± 0.1^d^** | 0.1 ± 0^bc^ | **0.1 ± 0.1^c^** | 0.1 ± 0^bc^ |
| *Clostridia* | *Unclass_Clostridiales* | OTU_1945 | 2 ± 1.6^a^ | 2.9 ± 4.8^a^ | 2.4 ± 1.4^a^ | 0.5 ± 0.1^a^ | 0.6 ± 0.2^a^ | 1.5 ± 1.4^a^ |
|  | *Unclass_Lachnospiraceae* | OTU_199 | 2.9 ± 0.7^c^ | 1.3 ± 0.4^a^ | 3.1 ± 0.6^c^ | 1.6 ± 0.3^ab^ | 3.5 ± 0.8^c^ | 2.4 ± 0.4^bc^ |
|  | *Unclass_Ruminococcaceae* | OTU_364 | 1.2 ± 0.1^b^ | 0.6 ± 0.2^a^ | 1.3 ± 0.1^b^ | 1.5 ± 0.3^b^ | 1.9 ± 0.6^b^ | 1.3 ± 0.4^b^ |
| *Cytophagia* | *Dyadobacter* | OTU_123 | 0.2 ± 0.3^a^ | 0.4 ± 0.7^a^ | 0.4 ± 0.2^ab^ | 0.7 ± 0.2^ab^ | 0.5 ± 0.3^ab^ | **1.4 ± 0.9^b^** |
|  | *Sporocytophaga* | OTU_2042 | 0 ± 0^a^ | 0 ± 0^a^ | **0.1 ± 0.1^b^** | 0 ± 0^a^ | 0 ± 0^a^ | 0 ± 0^a^ |
| *Flavobacteriia* | *Flavobacterium* | OTU_2086 | 0.1 ± 0^b^ | 0 ± 0^ab^ | 0 ± 0^ab^ | 0 ± 0^a^ | 0.1 ± 0^b^ | 0 ± 0^ab^ |
|  | *Unclass_Flavobacteriaceae* | OTU_2073 | 0.2 ± 0^ab^ | 0.1 ± 0.1^a^ | **0.6 ± 0.1^c^** | 0.3 ± 0.1^b^ | **0.5 ± 0.1^c^** | 0.2 ± 0^ab^ |
| *Gemmatimonadetes* | *Gemmatimonas* | OTU_265 | 0.5 ± 0.2^ab^ | 0.4 ± 0.1^a^ | **1.3 ± 0.2^d^** | 0.7 ± 0.1^bc^ | **1 ± 0.3^cd^** | 0.7 ± 0.1^bc^ |
|  |  | OTU_479 | 0.2 ± 0.1^a^ | 0.1 ± 0^a^ | 0.1 ± 0.1^a^ | 0.1 ± 0^a^ | 0.2 ± 0^a^ | 0.1 ± 0^a^ |
| *Nitrospira* | *Nitrospira* | OTU_2098 | 0.2 ± 0.1^ab^ | 0.1 ± 0.1^a^ | **0.6 ± 0.1^c^** | 0.2 ± 0.1^b^ | 0.3 ± 0.1^bc^ | 0.2 ± 0.1^b^ |
| *Sphingobacteriia* | *Chitinophaga* | OTU_201 | 1.4 ± 0.8^c^ | 0.4 ± 0.2^ac^ | 0.4 ± 0.3^ab^ | 1 ± 0.5^bc^ | 0.5 ± 0.5^ac^ | 0.2 ± 0.2^a^ |
|  |  | OTU_389 | 0.5 ± 0.1^b^ | 0.3 ± 0.1^ab^ | 0.2 ± 0.2^a^ | 0.3 ± 0.1^ab^ | 0.3 ± 0.1^ab^ | 0.3 ± 0.1^ab^ |
|  | *Ferruginibacter* | OTU_827 | 2.9 ± 1.2^c^ | 1.8 ± 0.7^c^ | 0.9 ± 0.3^ab^ | 0.7 ± 0^a^ | 1.9 ± 0.5^c^ | 1.6 ± 0.3^bc^ |
|  | *Flavisolibacter* | OTU_2041 | 0.1 ± 0.1^a^ | 0.1 ± 0.1^a^ | **0.4 ± 0.1^c^** | 0.1 ± 0^ab^ | **0.3 ± 0.1^c^** | **0.2 ± 0^bc^** |
|  |  | OTU_272 | 0 ± 0^a^ | 0.1 ± 0^a^ | **0.2 ± 0.1^c^** | **0.2 ± 0.1^bc^** | 0.1 ± 0.1^ab^ | 0.1 ± 0^ab^ |
|  | *Flavitalea* | OTU_91 | 0.6 ± 0.1^bc^ | 0.4 ± 0.2^ab^ | 0.3 ± 0.1^a^ | 0.3 ± 0^a^ | 0.8 ± 0.2^c^ | 0.6 ± 0.1^bc^ |
|  |  | OTU_481 | 0.1 ± 0^a^ | 0.1 ± 0.1^a^ | **0.4 ± 0.1^b^** | 0.1 ± 0^a^ | **0.4 ± 0.1^b^** | **0.3 ± 0.1^b^** |
|  | *Haliscomenobacter* | OTU_1954 | 1.8 ± 0.8^c^ | 2.2 ± 1.7^c^ | 0.3 ± 0.1^a^ | 0.2 ± 0^a^ | 1.3 ± 0.6^bc^ | 0.6 ± 0.1^ab^ |
|  | *Lacibacter* | OTU_2069 | 0 ± 0^a^ | 0 ± 0^a^ | **0.1 ± 0^c^** | 0 ± 0^ab^ | **0 ± 0^b^** | 0 ± 0^ab^ |
|  | *Mucilaginibacter* | OTU_583 | 0.7 ± 0.2^b^ | 0.6 ± 0.1^b^ | 0.5 ± 0.2^ab^ | 0.3 ± 0.1^a^ | 0.5 ± 0^ab^ | 0.7 ± 0.3^b^ |
|  |  | OTU_480 | 0.2 ± 0^c^ | 0.2 ± 0.1^c^ | 0.1 ± 0.1^ab^ | 0 ± 0^a^ | 0.1 ± 0^bc^ | 0.2 ± 0.1^c^ |
|  | *Niastella* | OTU_624 | 0.2 ± 0^a^ | **0.5 ± 0.3^b^** | **1.1 ± 0.3^d^** | **0.9 ± 0.3^cd^** | **0.5 ± 0.1^bc^** | **0.6 ± 0.2^bd^** |
|  | *Parasegetibacter* | OTU_1957 | 0 ± 0^a^ | 0 ± 0^a^ | **0.1 ± 0.1^bc^** | 0 ± 0^ab^ | **0.2 ± 0.1^c^** | 0.1 ± 0^ab^ |
|  | *Pedobacter* | OTU_183 | 1.1 ± 0.6^c^ | 1.1 ± 1.2^bc^ | 0.1 ± 0.1^a^ | 0.2 ± 0.1^ab^ | 0.3 ± 0.2^ab^ | 0.3 ± 0.1^ac^ |
|  |  | OTU_381 | 0.6 ± 0.1^b^ | 0.9 ± 0.8^b^ | 0.3 ± 0.2^ab^ | 0.1 ± 0^a^ | 0.7 ± 0.1^b^ | 0.7 ± 0.3^b^ |
|  |  | OTU_1943 | 0.1 ± 0^a^ | 0.1 ± 0^a^ | **0.2 ± 0.1^b^** | 0.1 ± 0^ab^ | **0.2 ± 0.1^b^** | **0.2 ± 0.1^b^** |
|  |  | OTU_559 | 0 ± 0^a^ | 0 ± 0^a^ | **0.3 ± 0.1^b^** | 0.1 ± 0^a^ | **0.3 ± 0.1^b^** | 0.1 ± 0.1^a^ |
|  | *Terrimonas* | OTU_444 | 0 ± 0^a^ | 0 ± 0^a^ | **0.6 ± 0.3^c^** | 0.1 ± 0^ab^ | **0.4 ± 0.1^c^** | **0.2 ± 0.1^b^** |
|  | *Unclass_Sphingobacteriales* | OTU_2087 | 0 ± 0^a^ | 0 ± 0^a^ | 0 ± 0^a^ | **0.1 ± 0^b^** | 0 ± 0^a^ | 0 ± 0^a^ |
| *Thermomicrobia* | *Sphaerobacter* | OTU_770 | 0 ± 0^ab^ | 0 ± 0^a^ | **0.1 ± 0^c^** | **0.1 ± 0^c^** | 0 ± 0^b^ | 0 ± 0^ab^ |
| *Unclass_Bacteria* | *Unclass_Bacteria* | OTU_682 | 0.5 ± 0.1^b^ | 0.2 ± 0.1^a^ | **0.9 ± 0.1^c^** | **0.9 ± 0.1^c^** | 0.4 ± 0.1^b^ | 0.3 ± 0.1^b^ |
| *Verrucomicrobiae* | *Luteolibacter* | OTU_168 | 0.1 ± 0^a^ | 0 ± 0^a^ | **0.5 ± 0.2^bc^** | **0.3 ± 0.1^b^** | **0.6 ± 0.3^c^** | **0.3 ± 0.1^bc^** |
|  | *Unclass_Verrucomicrobiaceae* | OTU_1360 | 0 ± 0^a^ | 0 ± 0^a^ | 0.1 ± 0^ab^ | 0.1 ± 0^ab^ | **0.1 ± 0.1^b^** | **0.1 ± 0^b^** |

**Table S4:** Relative abundances of all responding genera detected after two weeks after transplantation in response to the antagonist P142 or B63 and/or the pathogen *R. solanacearum* B3B in the rhizosphere of tomato. Numbers give the average percentage followed by ± standard deviation (n=4), treatments sharing the same letters are non-significantly different (*p*< 0.05, ANOVA with generalized linear model followed by Tukey honest significant detection test). Significant increases in abundance compared to TC are highlighted in green, while significant decreases are highlighted in red.
